# Assessment of halotolerant bacterial and fungal consortia for augmentation of wheat in saline soils

**DOI:** 10.3389/fmicb.2023.1207784

**Published:** 2023-06-30

**Authors:** Muhammad Usama Marghoob, Aniqa Nawaz, Muhammad Ahmad, Muhammad Qandeel Waheed, Muhammad Hassaan Khan, Muhammad Imtiaz, Ejaz ul Islam, Asma Imran, Fathia Mubeen

**Affiliations:** ^1^Soil and Environmental Biotechnology Division, National Institute for Biotechnology and Genetic Engineering, Constituent College of Pakistan Institute of Engineering and Applied Sciences (NIBGE-C, PIEAS), Islamabad, Pakistan; ^2^Plant Breeding and Genetic Division, Nuclear Institute for Agriculture and Biology (NIAB), Faisalabad, Pakistan; ^3^Department of Bioinformatics and Biotechnology, Government College University Faisalabad (GCUF), Faisalabad, Pakistan

**Keywords:** abiotic stress, salinity, halotolerant PGPR, plant-microbe interaction, root structure architecture

## Abstract

Adaptations of green technologies to counter abiotic stress, including salinity for crops like wheat by using halotolerant microbes, is a promising approach. The current study investigated 17 salt-affected agroecological zones from the Punjab and Sindh provinces of Pakistan to explore the potential of indigenous microbial flora, with their multiple biochemical characteristics in addition to plant growth promoting (PGP) traits, for enhanced wheat production in saline areas. Initially, 297 isolated pure bacterial colonies were screened for salt tolerance, biochemical, and PGP traits. Three bacterial strains belonging to *Pantoea* spp. and *Erwinia rhaphontici* with possession of multiple characteristics were selected for the development of the halotolerant bacterial consortium. Inoculation of two local wheat varieties, Faisalabad 2008 and Galaxy 2013, with the consortium for *in vitro* seed germination assay and sand microcosm experiments exhibited significant improvement of selected plant growth parameters like germination percentage and root structure. Two previously reported PGP fungal strains of *Trichoderma harzianum* and *T. viridae* were also used as fungal consortium separately for pot experiments and field trials. The pot experiments exhibited a positive correlation of consortia with metabolic *viz.* catalase, peroxidase, and proline and agronomical parameters including shoot length, dry weight, number of spikes, spike length, and 100 grain weight. To evaluate their performance under natural environmental conditions, field trials were conducted at three salt-affected sites. Agronomical attributes including days of flowering and maturity, flag leaf weight, length and width, shoot length, number of spikes, spike length, spike weight, number of seeds spike^−1^, 1,000 grain weight, and plot yield indicated the efficiency of these microbes to enhance wheat growth. Concisely, the bacterial consortium showed better performance and Faisalabad 2008 was a more resistant variety as compared to Galaxy 2013. Initial promising results indicate that further extensive research on indigenous microbes might lead to the development of Pakistan’s first saline-specific biofertilizers and sustainable eco-friendly agriculture practices.

## 1. Introduction

With the rapidly growing and evolving world, new problems, especially those linked to climate change, are emerging. For instance, the key requirement for increasing world population is the availability of sufficient food. However, anthropogenic activities along with other environmental issues are driving factors associated with food security. Collectively, these problems are not only deteriorating the planet but also pose a serious threat to agriculture (Nawaz A. et al., 2020).

Expanding salinization of agricultural lands, which is a global issue in general and serious threat to countries like Pakistan, needs special attention (Shah et al., 2021). Wheat (*Triticum aestivum*) is Pakistan’s main staple food and is considered the gold crop of the country (Nawaz A. et al., 2020; Azmat et al., 2021). In addition to many other major crops including rice, maize, etc., wheat also belongs to the group of plants known as “glycophytes” as it is susceptible to saline conditions (Uçarlı, 2020). Therefore, special emphasis is given to exploring options for mitigating the adverse effects of salinity on wheat. Currently, different approaches, e.g., physical and chemical applications, plant breeding, and molecular biology techniques, are being practiced and investigated to tackle this problem, but the outcomes are not satisfactory (Nouri et al., 2017; Ben-Laouane et al., 2020; Perez-Jimenez and Perez-Tornero, 2020; Rani et al., 2022). However, the use of microorganisms is considered to be a more efficient and ecofriendly approach for sustainable agriculture (Mukhtar et al., 2020; Nawaz A. et al., 2020; Marghoob et al., 2022).

Microbes are helping mankind even before their discovery. The microbial world plays a key role in mitigation of major problems. Exploiting the microbial flora, with plant growth promoting (PGP) characteristics, for improve plant growth and combating biotic and abiotic stresses is a well-known phenomenon (Nawaz A. et al., 2020; Cochard et al., 2022). These tiny creatures harbor genes that facilitate plant growth through activities including N_2_-fixation, P-solubilization, K-solubilization, siderophore and other phytohormones production, antimicrobial compounds, and induced systemic resistance against biotic and abiotic agents (Islam et al., 2016; Nawaz et al., 2021; Fatima et al., 2022).

In current era, exploiting the halotolerant indigenous microbial flora, for crop improvement in a saline environment, is a promising approach to address the adverse effects of salinity (Li et al., 2022). In addition to other PGP traits, some indigenous microbes from harsh environments also contain ACC-deaminase enzyme that positively regulates ethylene, produced excessively in plants under stress environments and these elevated levels are dangerous for plant health (Bouffaud et al., 2018; Ben-Laouane et al., 2020). Production of osmoprotectants like glycine betaine, proline, and ectoine, etc. also help both plants and microbes to adapt the harsh saline conditions (Chen et al., 2021). Production of some secondary metabolites like exopolysaccharides (EPS) and other enzymatic activities also leverage these microbes for better adaptation to harsh environments and to facilitate crop resilience against abiotic factors (Mishra et al., 2016; Batool et al., 2017; Ahmad M. et al., 2022). Production of EPS not only helps these microbes in adaptations but also improves soil properties and helps in Na^+^ scavenging (Egamberdieva et al., 2019). In addition to other PGP activities, these microbes also have the potential to facilitate plant growth under saline environment by enhancing plant metabolic activities like Catalase, Peroxidase, superoxide dismutase and production of osmoprotectants (Sekar et al., 2019; Nawaz M. S. et al., 2020).

Interestingly, PGP-related research has mainly focused on bacteria and mycorrhiza previously; however, recent findings unleashed the hidden PGP potential of some fungal strains, particularly from the genus *Trichoderma*, in addition to their role as a biocontrol agent against phytopathogens (Martinez-Medina et al., 2014; Faraz et al., 2020; Sánchez-Montesinos et al., 2020). Strains belonging to *Trichoderma* are not only important to improving plant growth by their involvement in nutrient uptake, improved soil physiochemical properties, phytohormone production, and combating phytopathogens, but they also have a promising role in alleviating biotic stresses, including salinity (Sánchez-Montesinos et al., 2020).

Similarly, the application of multiple strains possessing a diverse set of PGP characteristics, in the form of a consortium, under field conditions is considered a practical approach to minimizing the adverse effects of environmental and seasonal variations on the survival and functional potential of individual microbes (Etesami and Maheshwari, 2018). Various studies showed that the efficacy of microbes used in this form of consortium perform better than inoculation with individual strains (Sekar et al., 2016; Devi et al., 2022; Kaur et al., 2023).

The current study was designed to focus on the saline agricultural zones of the Indus Basin region from the provinces of Punjab and Sindh, Pakistan (the breadbasket of the country), where low wheat production is directly linked to the expansion of salinity in association with other factors (Syed et al., 2020). Moreover, most of the areas in these vicinities have remained unexplored in terms of microbial flora and their PGP traits. The present study hypothesized that isolation and characterization of bacterial strains from these soils would facilitate the finding of the indigenous microbial flora with PGP traits that could be used in the future for the formulation of saline-specific biofertilizers, and outcomes of the study would not only help in wheat growth improvement under saline conditions but would also lead to less dependency on chemical fertilizers.

## 2. Materials and methods

### 2.1. Isolation and characterization of PGP bacterial strains

#### 2.1.1. Soil samples collection

For the isolation of efficient plant growth-promoting halotolerant bacterial strains, rhizospheric and bulk soil samples were collected from 17 salt-affected agricultural lands located in the Punjab and Sindh provinces of the Indus Basin region, Pakistan. Wheat is cultivated in these areas during winter with other rotational summer crops. A list of sample sites is provided in Supplementary File 1, Table 1. Three soil samples were collected from a depth of 4–6 inches using a sterile soil probe from each location, and they were then homogenized and carefully transferred to the soil physiology lab, National Institute for Biotechnology and Genetic Engineering (NIBGE), Faisalabad, in sterile bags and stored at 4°C. Basic chemical properties, electrical conductivity (EC) and pH of soil, were determined for each sample in order to estimate the salinity level to evaluate these samples for isolation of potential halotolerant PGP microbes.

#### 2.1.2. Selection of halotolerant bacterial isolates

For bacterial strain isolations, serial dilution method was opted. Briefly, 1 g of homogenized soil sample from each site was added to 9 mL normal saline (NS) solution (0.9% NaCl) and mixed thoroughly to make a 10^−1^ dilution. For 10^−2^ dilution, 1 mL from 10^−1^ was added to 9 ml NS and was mixed properly. Serial dilutions were prepared up to 10^−5^ using this method, and 10^−3^ and 10^−5^ dilutions were used for spreading on LB agar and LB agar plates supplemented with 1% NaCl in triplicates. Plates were incubated at 28 ± 2 ͦ C for 24–48 h.

Plates were observed for bacterial growth, and 297 bacterial isolates were selected to determine their minimal inhibitory concentration (MIC) for NaCl. LB agar plates supplemented with NaCl in a gradually increasing order (0.5, 1, 5, 7, and 10%) were used for this purpose. Plates were incubated at 28 ± 2 ͦ C for 24–48 h.

#### 2.1.3. Screening for plant growth promoting characteristics

Only those bacterial strains that were able to withstand MIC (7% and/ or 10%) were selected for assessment of PGP characteristics like P-, Zn- and K-solubilization, IAA and siderophore production, and ACC deaminase activity, etc. Briefly, P- solubilization activity was checked using Pikovskaya’s medium by methods described earlier (Pikovskaya, 1948) both qualitatively and quantitatively. IAA production was evaluated by following the protocol of Gordon and Weber (1951) on tryptophan-supplemented media. K- solubilization characteristics were determined by observing hollow zones on Alexandrov’s medium (Boubekri et al., 2021). Siderophore production was detected by using the standard procedure described by Schwyn and Neilands (1987). ACC deaminase activity was assessed as described by Kumar et al. (2021).

### 2.2. Detection of selected biochemical pathways

Different biochemical pathways and metabolites especially linked with the survival of microorganisms under challenging environmental conditions (e.g., exopolysaccharides, amylase, hydrogen cyanide, catalase, and peroxidase production) were also investigated. Briefly, the ability of exopolysaccharide (EPS) production for the selected halotolerant strains was evaluated by the method described by Batool et al. (2017). A starch hydrolysis test (Peltier and Beckord, 1945) was carried out for the detection of α-amylase enzyme production. Hydrogen Cyanide (HCN) production, an efficient strategy of microbes against potential pathogens, was detected by the method described earlier in the literature (Nandi et al., 2017). Catalysis activity for toxic hydrogen peroxide (H_2_O_2_) conversion into water and Oxygen by bacteria using catalase enzymes was determined by the previously described method (Attia et al., 2022).

### 2.3. Molecular identification of selected strains

Overnight-grown bacterial cultures on LB broth were used for bacterial DNA isolation and amplification through PCR. Bacterial genomic DNA was extracted by using the GeneJET Genomic DNA Purification Kit (Thermo Scientific, Carlsbad, CA, United States of America) as per the manufacturer’s instruction. DNA was quantified using a Nanodrop spectrophotometer (Thermo Scientific, Waltham, MA, United States of America). Universal primer set fD1 and rD1 and the optimized PCR profile (Supplementary File 1, Table 2) were used for bacterial 16S rRNA gene amplification (Fatima et al., 2022). PCR products were confirmed on 1% agarose gel before shipping to Macrogen, Inc. South Korea, for sequencing.

The 16S rRNA gene partial sequences of UM16, UM 58, and UM 83 were compared with available sequences in the GenBank database using the NCBI Basic Local Alignment Search Tool (BLAST) and were submitted to the NCBI gene bank repository, and accession numbers were obtained.

A neighbor-joining phylogenetic tree using MEGA software v.11.0 with 10,000 bootstrap values, where bootstrap values are shown in figure, was constructed.

### 2.4. Strains compatibility assessment

The cross-streak method on LB media was used to check the strains compatibility in the possible consortium (Fatima et al., 2022).

### 2.5. *In vitro* assessment for improvement in wheat growth parameters under saline stress

To evaluate the response of wheat germination in saline environments at different NaCl concentrations in the presence of PGP bacteria, three selected bacterial strains, UM 16, UM 58, and UM 83, were used in the form of the consortium to conduct plate assay for seed germination.

#### 2.5.1. Plate assay for seed germination

Seeds of two wheat varieties, Galaxy 2013 (V1) and Faisalabad 2008 (V2), were collected from Ayub Agriculture Research Institute (AARI), Faisalabad, and were used for the experimentation.

Healthy seeds of both varieties were surface sterilized using sodium hypochlorite (2%) and autoclaved water (Cheng et al., 1997). Seed priming of glycophytes with a saline solution of lower concentrations (halopriming) might also improve seed germination (Uçarlı, 2020); therefore, seeds soaked in sterilized water (T1) and 0.2 mM NaCl solution (T2) were used as controls separately. For seed biopriming, seeds were soaked for 30–40 min in 24-h old LB-broth culture with an adjusted OD of 0.1 at 600 nm of consortium for bacterial treatment (T3).

Seeds were transferred to Petri dishes containing 1% water agar [and 0, 50, 100, and 150 mM of NaCl for each treatment, where; 10 mM ~ 1dSm^−1^ (Rahnama et al., 2011)] aseptically in such a manner that each plate carried 6–7 seeds for each treatment in triplicates. Plates were incubated at 24 ± 4°C for 7 days in a growth chamber and were observed daily for seed germination.

The germination percentage (GP) was calculated by using the following formula (Manmathan and Lapitan, 2013):


GP=germinated seeds/total seeds×100

### 2.6. Plant root structure studies

In order to understand the halotropism response, seeds of two wheat varieties, Faisalabad 2008 and Galaxy 2013, were used to visualize the effect of inoculation on root growth and architecture under NaCl stress. Seeds were sterilized and grown on 1% agar plates as described in the previous section. Seeds were transferred to pots containing sterilized sand after 7 days of germination and were transferred to the greenhouse. Sterilized tap water, NaCl solutions of selected concentrations (0, 20, 40, 60, and 80 mM), and Hoagland solution (Hoagland and Arnon, 1950; Spehia et al., 2018) with 80 and 100% concentrations were used for control and treatments as per requirement in the following manner; T1: C100 (Hoagland 100% conc.), T2: C80 (Hoagland 80% conc.), and T3: TB (bacterial consortium and Hoagland 80% conc.).

Plants were harvested after 18–20 days and roots were carefully collected, washed, and properly labeled. Three plants from each treatment were used for root structure parameters studies. A Rhizoscanner (Epson photo scanner V700) installed with WinRHIZO (Regent Int. Co., Ltd.) was used to study selected parameters including root length (cm), surface area (cm^2^), the average diameter (mm), root volume (cm^3^), and root tips.

### 2.7. Revival of PGP fungal strains

Two previously reported PGP fungal strains, *Trichoderma harzianum* and *T. viridae* (Faraz et al., 2020), were collected from the National Bioresource Collection Center (NBRC), NIBGE, Pakistan, and revived on PDA media. Spores were placed on PDA plates and incubated at 28 ± 2 ͦ C for 5–7 days.

### 2.8. Preparation of bacterial and fungal bioformulations

Three selected bacterial strains, UM 16, UM 58, and UM 83, and fungal strains *Trichoderma harzianum* and *T. viridae* were used for the development of bacterial and fungal consortium separately for pot experiments and field trials. Overnight LB broth cultures of bacteria were mixed equally after calculating the cell density, and the final concentration of the consortium was 10^8^ CFU ml^−1^. Fungal cultures of 5–7 days, grown in PD broth, were used, and the final concentration of spores was adjusted to 10^8^ ml^−1^. Previously characterized sterilized filter mud was used as carrier material (Suleman et al., 2018).

### 2.9. Pot experiment under control and saline conditions

In order to evaluate the wheat growth response to fungal and bacterial consortia at different NaCl concentrations under natural environmental conditions, a pot experiment was conducted (Dec 2017-April 2018). Earthen pots containing 11–12 Kg homogenized sterile soil were used and three different salinity levels (2, 6, and 10 dSm^−1^) were attained by using NaCl solutions of different concentrations and monitoring of soil periodically with an EC meter to maintain maximum homogeneity. Two controls, T1: 100% farmer recommended dose (FRD) of fertilizer and T2: 80% FRD were used, and bacterial (T3) and fungal (T4) consortia with 80% FRD were used separately to evaluate their effects on wheat at different salinity levels. The recommended dose of chemical fertilizers applied in this region is N:P; 15:100 Kg ha^−1^. The required FRD of fertilizers in the form of Urea and DAP was applied at the time of sowing and first and second irrigation. The wheat varieties Faisalabad 2008 and Galaxy 2013 were used, and a randomized complete block design (RCBD) with triplicates for each treatment was opted for. In each pot, 10–12 seeds were planted.

#### 2.9.1. Agronomical and biochemical parameters

At the thinning stage, five/six plants were uprooted from each pot, and three plants were selected randomly for the detection of selected metabolic/enzymatic activities associated with salinity stress (catalase, peroxidase, and free proline content) using previously optimized protocols (Nawaz A. et al., 2020). At the maturity stage, selected agronomical parameters *viz.* shoot length, shoot dry weight, number of spikes per plant, spike length, and 100 grain weight were calculated for three randomly selected plants from each replicate.

### 2.10. Field trials

In order to evaluate the response of selected PGP bacterial and fungal consortia under natural saline conditions on wheat cultivation, trials were conducted at three different sites *viz.* the Soil Salinity Research Institute (SSRI), Pindi Bhattiyan (2018–2019) (S1); a salt-affected farmer’s field in Mouza sheikhana, Jhang (2020–2021) (S2); and Biosaline Research Station (BSRS II), Pakka Anna (2018–2019) (S3).

#### 2.10.1. Soil chemical properties

The soil’s basic chemical properties were determined for each site to estimate the salinity level and other nutritional factors before experimentation. Standard procedures were followed to determine electrical conductivity (EC), pH, soil organic matter (SOM), total nitrogen (N), available phosphorous (P), and ionic concentrations (Cl^−^, Na^+^, K^+^; Walkley and Black, 1934; Olsen, 1954; Richard, 1954; Imran et al., 2021).

#### 2.10.2. Experimental design

Briefly, two local wheat varieties, Faisalabad 2008 and Galaxy 2013, were selected, and healthy untreated seeds were used for two controls, one with 100% FRD of fertilizer (T1) and the second with 80% FRD (T2). Seeds, bio-primed with bacterial (T3) and fungal (T4) consortia separately as bioinoculants, were used for treatments in these trials, and a randomized complete block design (RCBD) with plot size (2×5 m^2^) in triplicates for each location was implemented.

#### 2.10.3. Plant growth parameters

Different agronomical parameters like days of flowering, days of maturity, flag leaf width, flag leaf length, flag leaf weight, plant height, number of spikes per plant, spike length with awns, spike weight, number of grains per spike, 1,000 grain weight, and total yield per plot were calculated to estimate the effects of formulations.

### 2.11. Statistical analyses

For the data analysis of the pot experiment, the parameters were assessed as mean ± S.E (standard error) followed by Dunnett’s tests and Tukey’s HSD tests for comparison with control (i.e., FDR 100%) individually and for intra-dosage comparison, respectively. All the statistical analyses were made using JMP v.11 (SAS, Cary, NC). The graphs were designed using Microsoft Excel v.2019.0. While for the field data, all the analyses were made in R. software v.4.2.1. for two-way ANOVA and combined correlation using tidyverse, AICcmodavg, and corrplot R packages. Boxplots and correlation matrixes were visualized using ggplot2 and ggthemes packages.

## 3. Results

### 3.1. Basic chemical properties of soil samples

Data for the ECs and pHs of all 17 samples is provided in Supplementary File 1, Table 1. Briefly, all soils were saline, ranging from moderately (4–8 dSm^−1^) to very strongly saline (>16) as per criteria described by Abrol et al. (1988). The lowest EC (6.08 ± 0.56) was recorded for samples collected from Choa-Saidan Shah-Kallar Kahar Road, Punjab (30.4319, 71.9958), and the highest (35.34 ± 1.02) was for samples from Bhago Bhutto, Dhairki, Sindh (28.0403, 69.6561). Soil pH for samples ranged from neutral to alkaline, and the lowest pH (7.23 ± 0.04) was observed for samples from Talaganag-Kallar Kahar Road, Punjab (32.7863, 72.6968) and the highest (9.02 ± 0.082) for Choa-Saidan Shah- Kallar Kahar Road, Punjab (32.7485, 72.7574).

### 3.2. Isolation and MIC determination for salt tolerance:

A total of 297 salt-tolerant strains were isolated from 17 sites in Punjab and Sindh (Supplementary File 2, Table 1). Their MIC (minimal inhibitory concentration) was determined, and 141 bacterial isolates that were able to withstand 7% and/ or 10% NaCl concentration were screened to assess plant growth-promoting characteristics (Supplementary File 2, Table 2).

### 3.3. Screening and selection of bacterial strains for bacterial consortia based on PGP activities and biochemical characterization

Details of selected PGP and biochemical characteristics of 141 halotolerant bacteria are provided in Supplementary File 2, Table 3. Many strains showed possession of multiple characteristics, and three strains UM 16, UM 58, and UM 83 were shortlisted to evaluate their performance on wheat growth under saline conditions. Briefly, these strains were selected on the basis of their overall PGP and biochemical properties. All three strains were able to solubilize the P, K, and Zn. None of the isolates were able to produce IAA as no color change was observed after the addition of Salkowski reagent. All three strains were able to produce siderophore, and average activity was recorded on CAS agar plates. Bacterial strains UM 16, UM 58, and UM 83 were catalase positive and oxidase negative. All strains were positive for EPS production and Amylase activity. No color change in media indicated the absence of HCN production for all three strains. ACC deaminase activity was detected only for UM 16. A summary of PGP traits including solubilization indices for P, K, and Zn, and selected biochemical characterization of three selected strains is provided in Table 1.

**Table 1:** Assessment of plant growth promoting and other selected biochemical characteristics of three bacterial strains used in consortium for evaluation of wheat growth under saline environments.

	Solubilization Index			Production				Other metabolic/ enzymatic activities			
	P	Zn	K	IAA	EPS	HCN	Siderophore	ACC deaminase	Catalase	Oxidase	Amylase
UM 16	3.33	3.42	4	−	+	−	+	+	+	−	+
UM 58	2.30	3.75	4.75	−	+	−	+	−	+	−	+
UM 83	1.28	2.33	4.75	−	+	−	+	−	+	−	+

Where, P = Phosphorus, Zn = Zinc, K = Potassium, IAA = Indole acetic acid, EPS = Exopolysaccharides, HCN = Hydrogen cyanide and “+” is an indication of presence while “−” indicates absence or no activity.

### 3.4. Molecular identification

**Figure 1:**
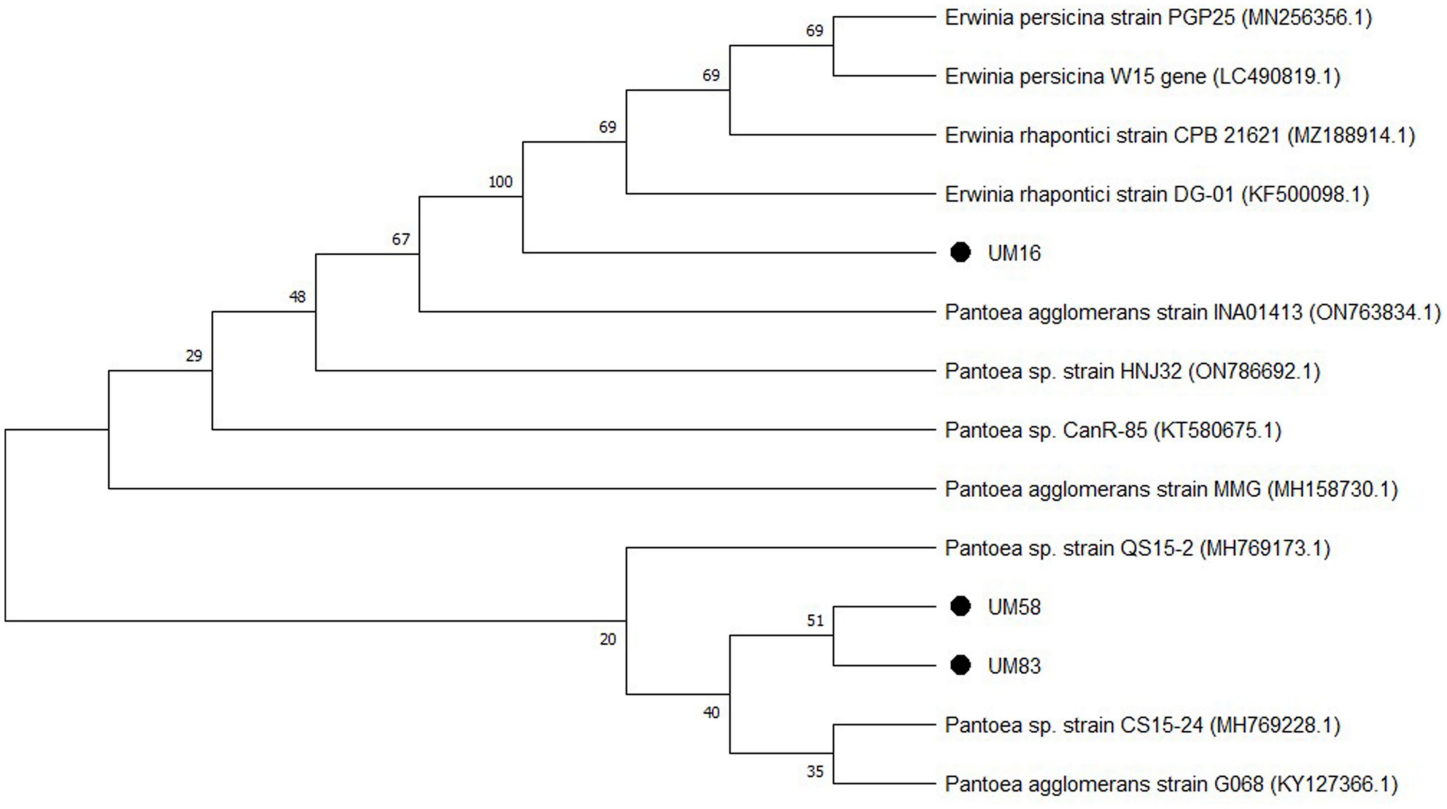
Phylogenetic tree of bacterial strains UM 16, UM 58, and UM 83, constructed using the neighbour-joining (NJ) method where >20% bootstrap values (10000) are shown at the nodes.

Bacterial strain UM 16 showed 99.76% similarity with the *Erwinia rhapontici* strain, strain UM 58 was identified as *Pantoea agglomerans* based on 99.61% similarity with the *P. agglomerans* strain submitted in the gene bank, while strain UM 83 was classified as *Pantoea* sp. on the basis of 97.01% similarity. The phylogenetic tree (Figure 1) indicated their origin, and the accession numbers obtained for UM 16, UM 58, and UM 83 are OQ318271, OQ318275, and OQ318278, respectively.

### 3.5. Strains compatibility assessment

No strain hindered the growth of other strains, and hence they were all considered compatible with each other for the formulation of consortium.

### 3.6. Plate assay for seed germination

**Figure 2:**
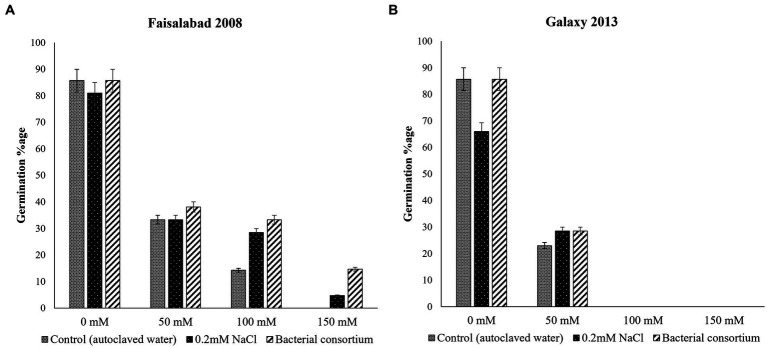
Effects of seed priming with autoclaved water, 0.2 mM NaCl solution, and bacterial consortium on seed germination percentage of wheat varieties **(A)** Faisalabad 2008 and **(B)** Galaxy 2013 at 0, 50, 100, and 150, mM concentrations of NaCl.

The effects of seed priming with water, 0.2 mM NaCl solution, and bacterial treatment on seed germination of both wheat varieties at different NaCl concentration is illustrated in Figure 2; Table 2. Overall, the germination percentage was better for Faisalabad 2008 under saline conditions as compared to Galaxy 2013, and the bacterial consortium improved the germination rate under saline conditions for both varieties. Seeds treated with 0.2 mM NaCl also showed relatively better performances than the control (T1). No seed germination was observed at 100 and 150 mM NaCl concentration in the case of Galaxy 2013, while only 4.7% seed germination was observed for the Faisalabad 2008 variety in the case of 0.2 mM NaCl pretreated seeds at 150 mM NaCl concentration and 14.2% for the bacterial consortium.

**Table 2:** Seed germination percentage of wheat varieties; Faisalabad 2008 and Galaxy 2013 at four different concentrations of NaCl, primed with autoclaved water, 0.2 mM NaCl solution, and bacterial consortium.

NaCl concentration	Seed germination percentage (%)					
	Faisalabad 2008			Galaxy 2013		
	Control (autoclaved water)	0.2 mM NaCl	Bacterial consortium	Control (autoclaved water)	0.2 mM NaCl	Bacterial consortium
0 mM	85.7	80.95	85.7	85.70	66.00	85.7
50 mM	33.33	33.33	38.1	23	28.5	28.5
100 mM	14.28	28.5	33.33	0	0	0
150 mM	0	4.7	14.7	0	0	0

### 3.7. Rhizoscanning for plant root architecture studies

**Figure 3:**
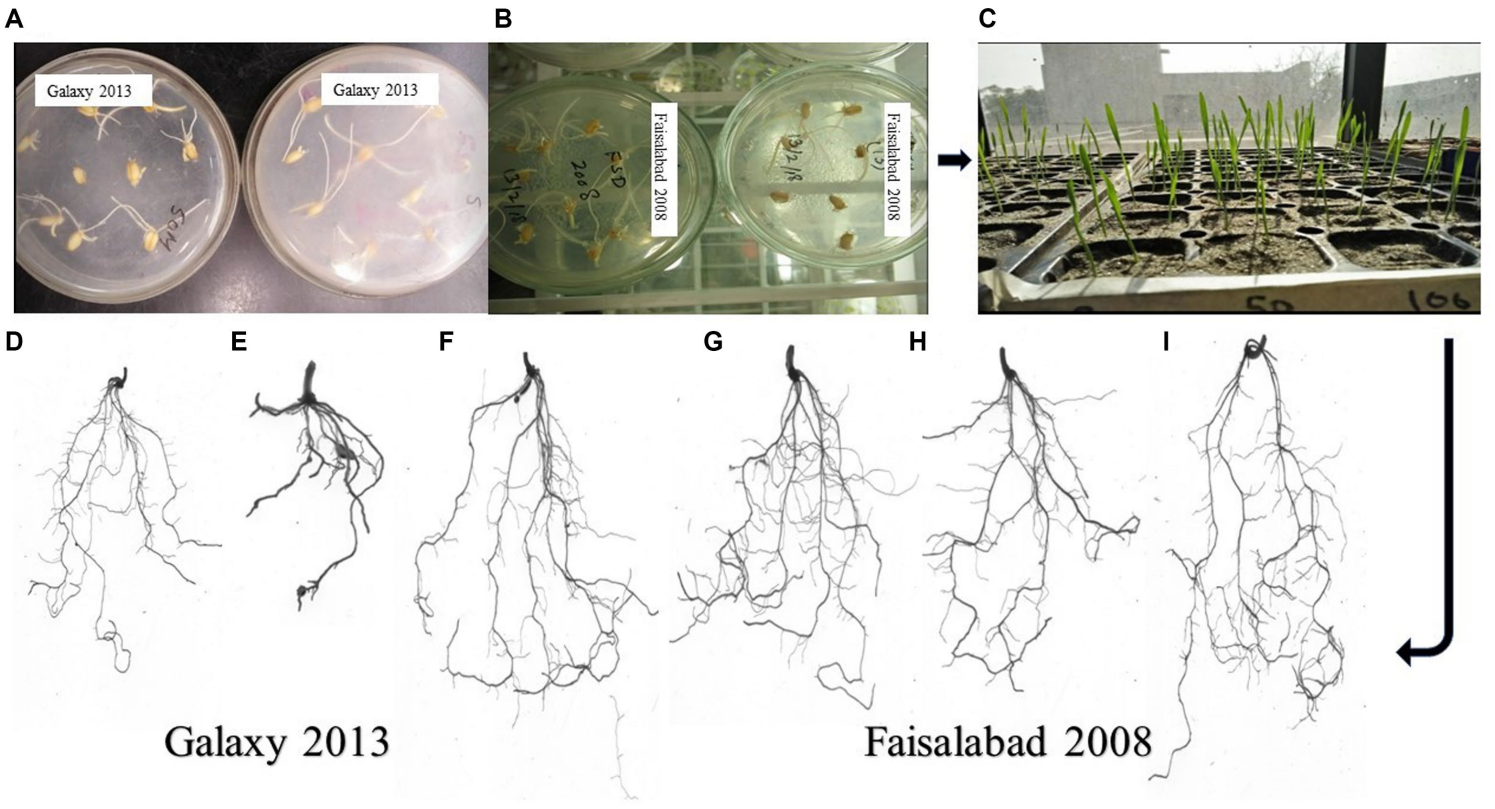
Root Architecture studies of two wheat varieties using a plate germination assay, where **(A)** Galaxy 2013 and **(B)** Faisalabad 2008 showed seed germination on 1% agar plates with 40 and 60 mM NaCl, respectively, and **(C)** plant growth in sterilized sand pots at different NaCl concentrations. Differences in root structure for Galaxy 2013 at 40 mM **(D)** 100% Hoagland concentration, **(E)** 80% Hoagland **(F)** Bacterial consortium, and for Faisalabad 2008 at 60 mM **(G)** 100% Hoagland concentration **(H)** 80% Hoagland **(I)** Bacterial consortium.

**Figure 4:**
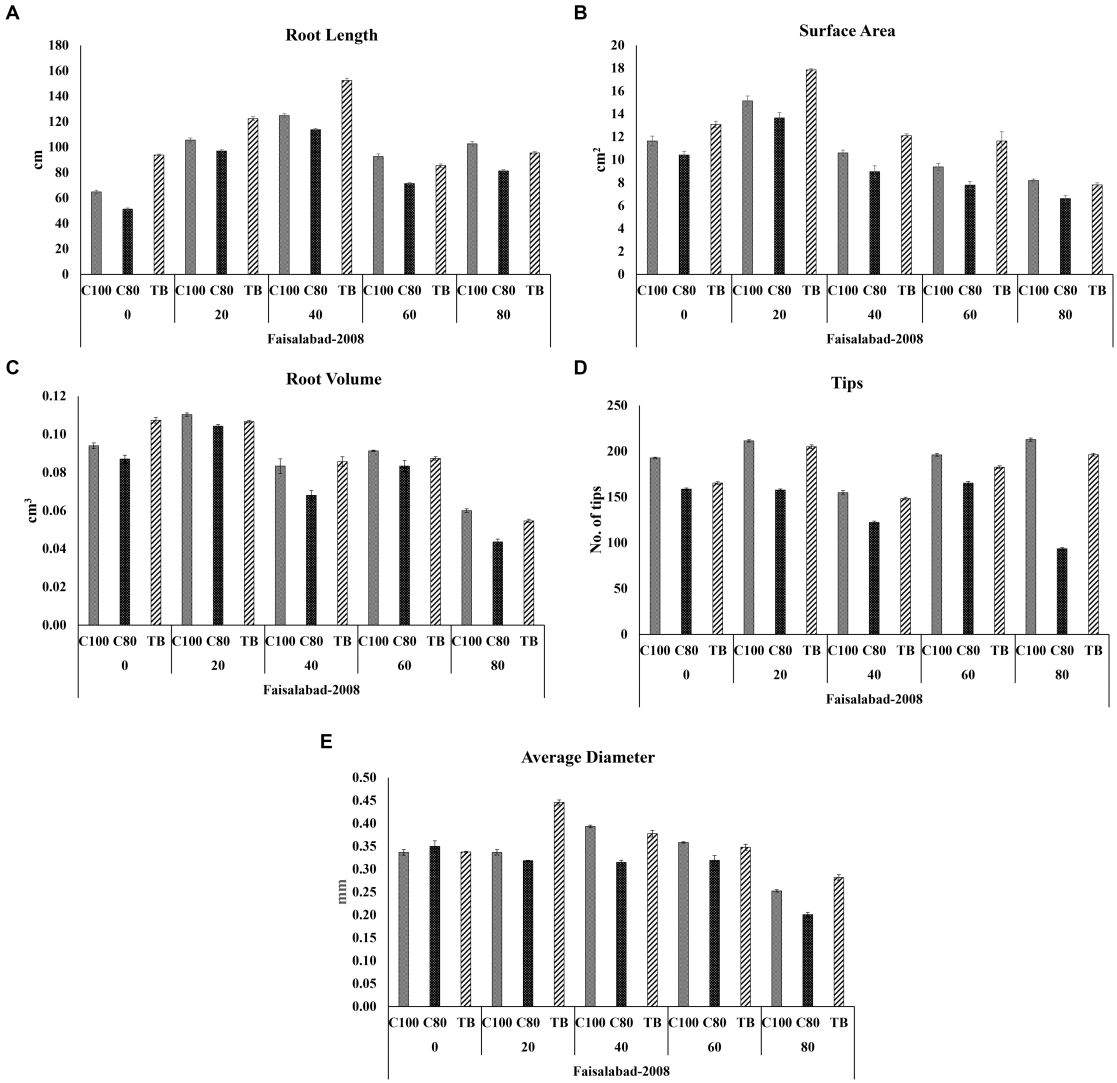
Effects of different concentrations of Hoagland solution C100 = 100%; C80 = 80% and BT = (bacterial consortium and 80% Hoagland solution) on selected root structure traits **(A)** Root length; **(B)** Surface area; **(C)** Root volume; **(D)** Tips **(E)** Average diameter, of wheat variety Faisalabad 2008 at 0, 20, 40, 60, and 80 mM NaCl concentrations where the bar indicates the standard error when n = 3.

**Figure 5:**
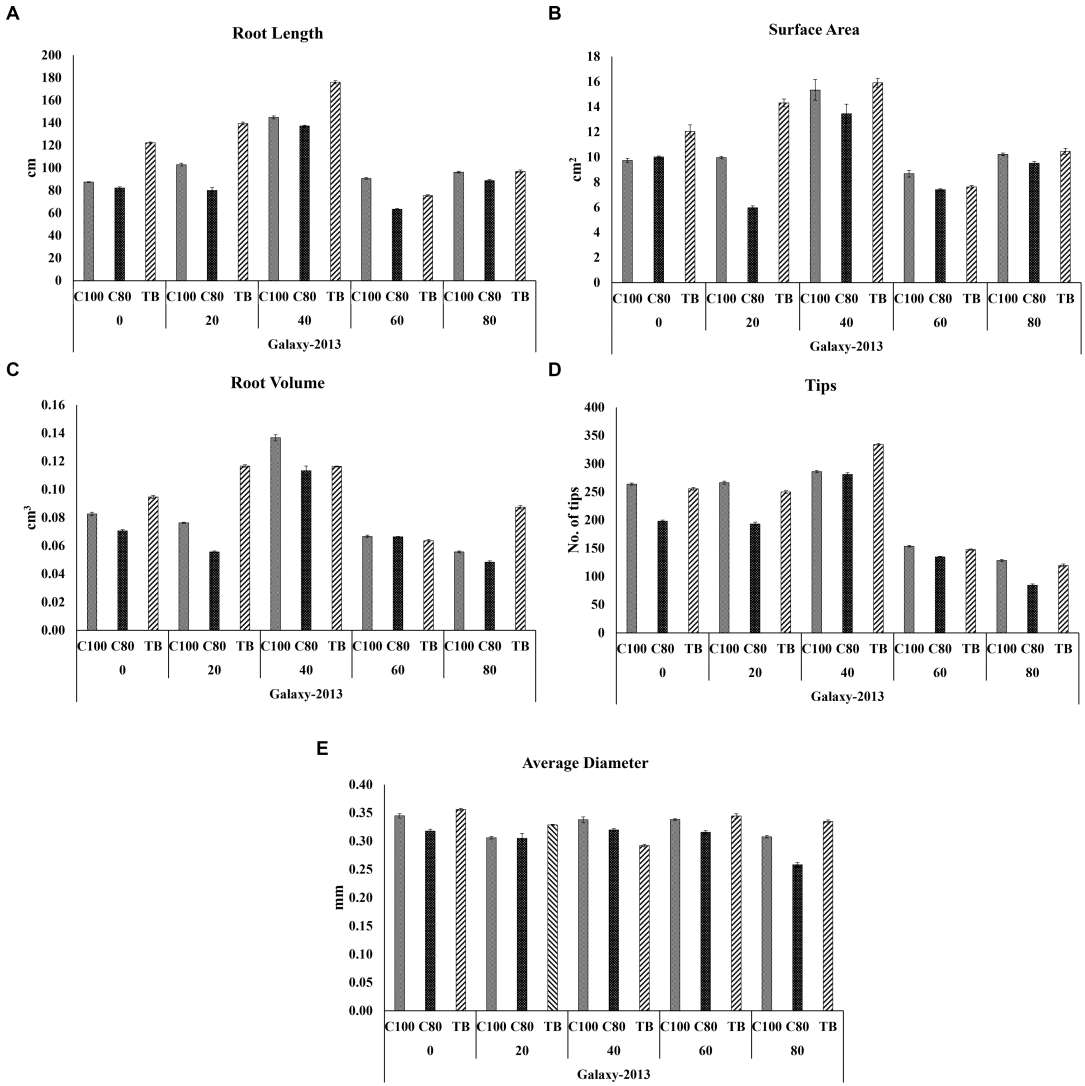
Effects of different concentrations of Hoagland solution C100 = 100%; C80 = 80% and BT = (bacterial consortium and 80% Hoagland solution) on selected root structure traits **(A)** Root length; **(B)** Surface area; **(C)** Root volume; **(D)** Tips **(E)** Average diameter, of the wheat variety Galaxy 2013 at 0, 20, 40, 60, and 80 mM NaCl concentrations where the bar indicates a standard error when n = 3.

Root rhizoscanning was conducted for two wheat varieties, Faisalabad 2008 and Galaxy 2013, at different salinity levels to observe the impact of bacterial treatment on various root parameters, including length and morphology. The plants treated with bacteria in both varieties exhibited significantly greater root length, surface area, and average root diameter compared to the non-treated plants across all salinity levels. The highest values for root length, surface area, and average root diameter were observed at 20 and 40 mM NaCl concentrations, ranging from 122 to 176 cm in length, 12 to 27cm^2^ in surface area, and 0.37 to 0.44 mm in root diameter. The maximum root volume and number of root tips were recorded at 0 and 20 mM NaCl concentrations, measuring 0.117 to 0.116cm^3^ and 165 to 334, respectively, as shown in Figures 3–5; Supplementary File 1, Tables 3, 4. Overall, all the seedlings treated with bacteria displayed superior performance compared to the control group (C2) that received 80% strength of Hoagland solution.

### 3.8. Pot experiment under control and saline conditions

The pot experiment was carried out to assess the five agronomical (shoot length and dry weight, number and length of spike, and 100 grain weight) and metabolic (catalases, peroxidases, and proline) parameters using two wheat varieties at three NaCl salinity levels. For the statistical significance, the trial was assessed using Dunnett’s test (*p* < 0.05) which correspondences to the control (FRD 100%) at each salinity level per treatment while the significance across the test salinity levels was assessed using ANOVA followed by Tukey’s HSD test (p < 0.05).

**Figure 6:**
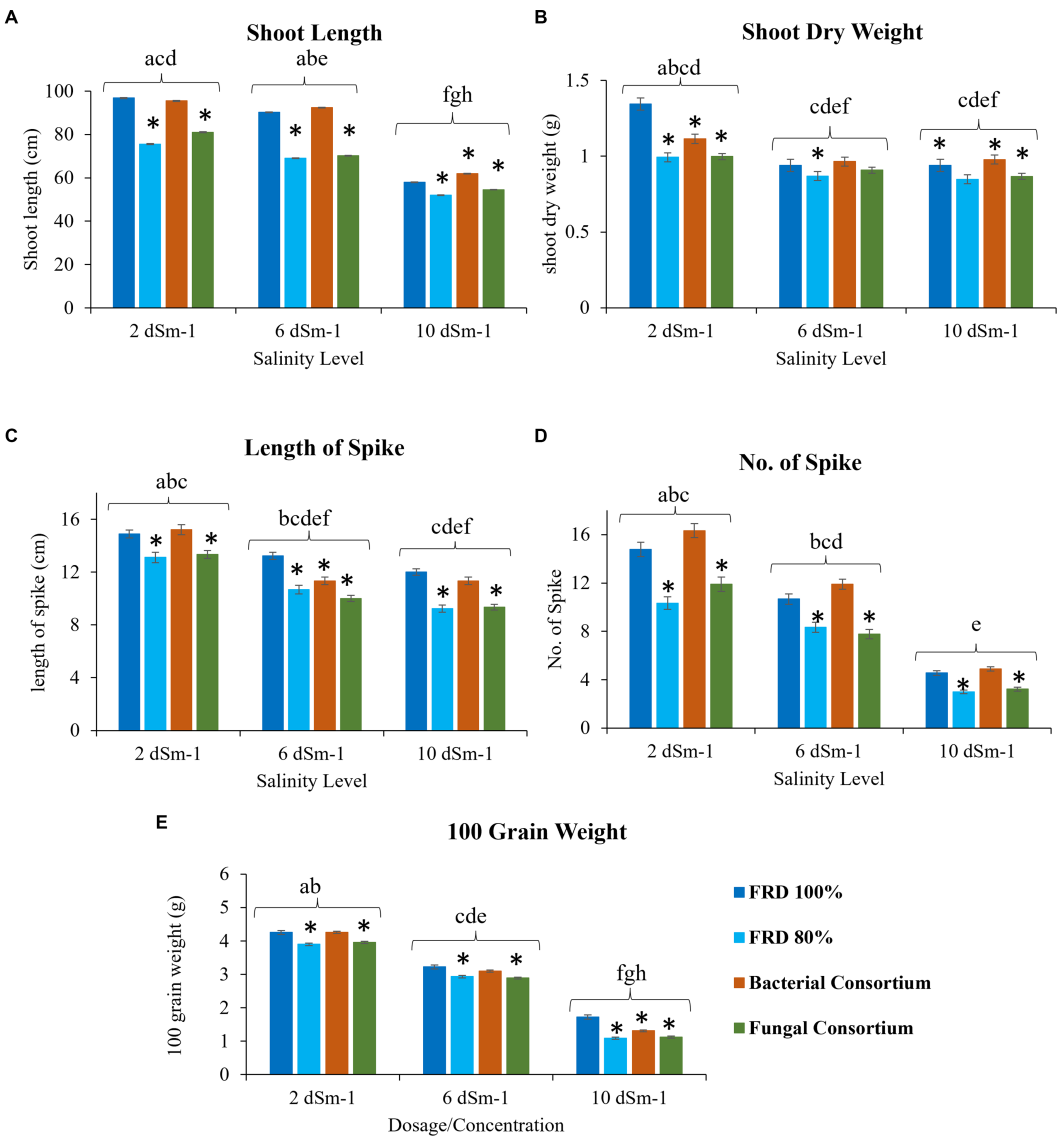
Effect of reduced fertilizer: 80% FRD and bacterial and fungal inoculation on agronomical parameters **(A)** Shoot length **(B)** shoot dry weight **(C)** Length of spike **(D)** No. of Spike **(E)** 100 grain weight; at different salinity levels for the wheat variety Faisalabad 2008 in pot experiment where bars indicate the Mean ± Standard Error of N = 3, n = 9; *p < 0.05, Dunnett’s test relative to control; FRD 100%. Different letters indicate *p* < 0.05, ANOVA followed by Tukey’s HSD test across the test dosage.

**Figure 7:**
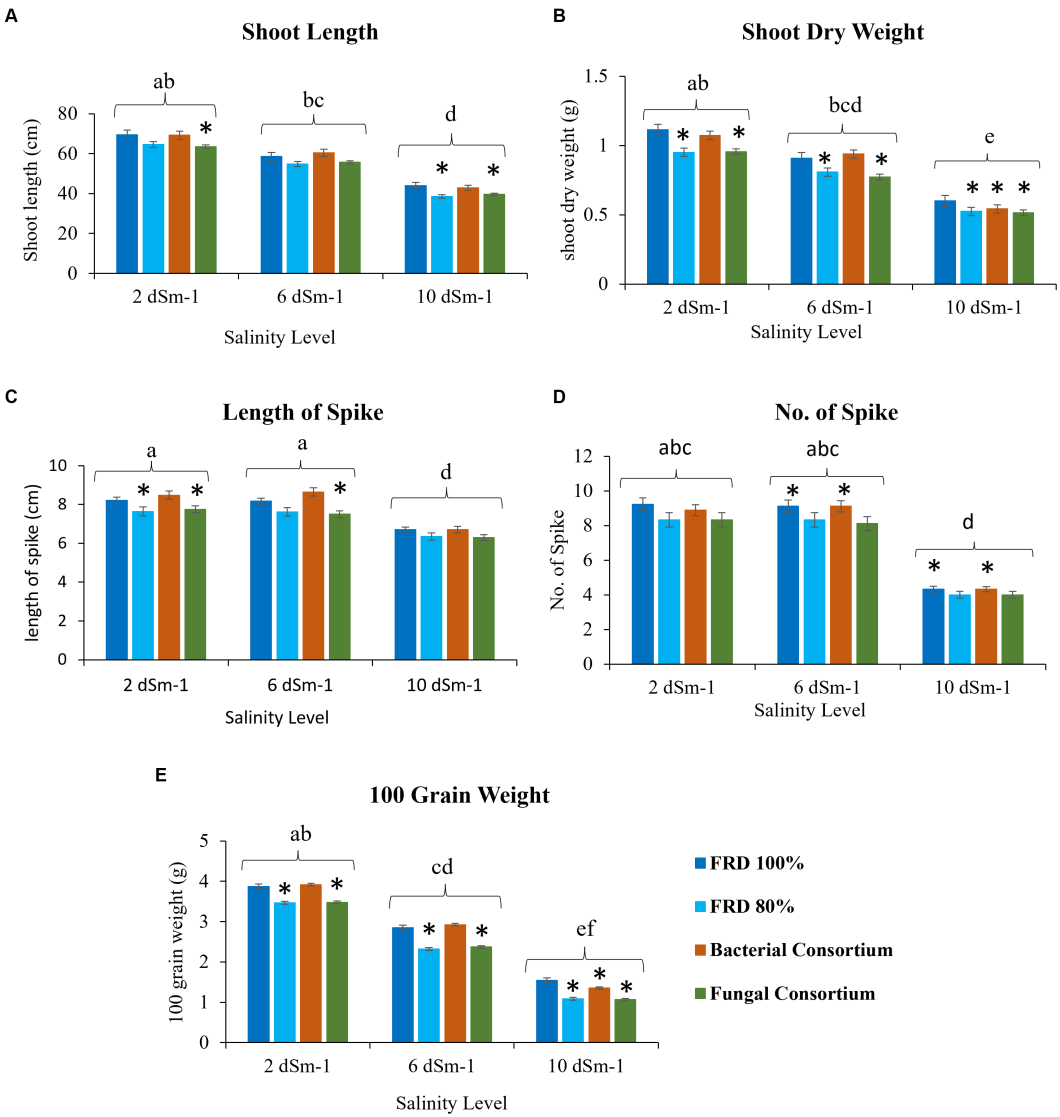
Effect of reduced fertilizer: 80% FRD and bacterial and fungal inoculation on agronomical parameters **(A)** Shoot length **(B)** shoot dry weight **(C)** Length of spike **(D)** No. of Spike **(E)** 100 grain weight; at different salinity levels for the wheat variety Galaxy 2013 in pot experiment where bars indicate the Mean ± Standard Error of N = 3, n = 9; *p < 0.05, Dunnett’s test relative to control; FRD 100%. Different letters indicate *p* < 0.05, ANOVA followed by Tukey’s HSD test across the test dosage.

**Figure 8:**
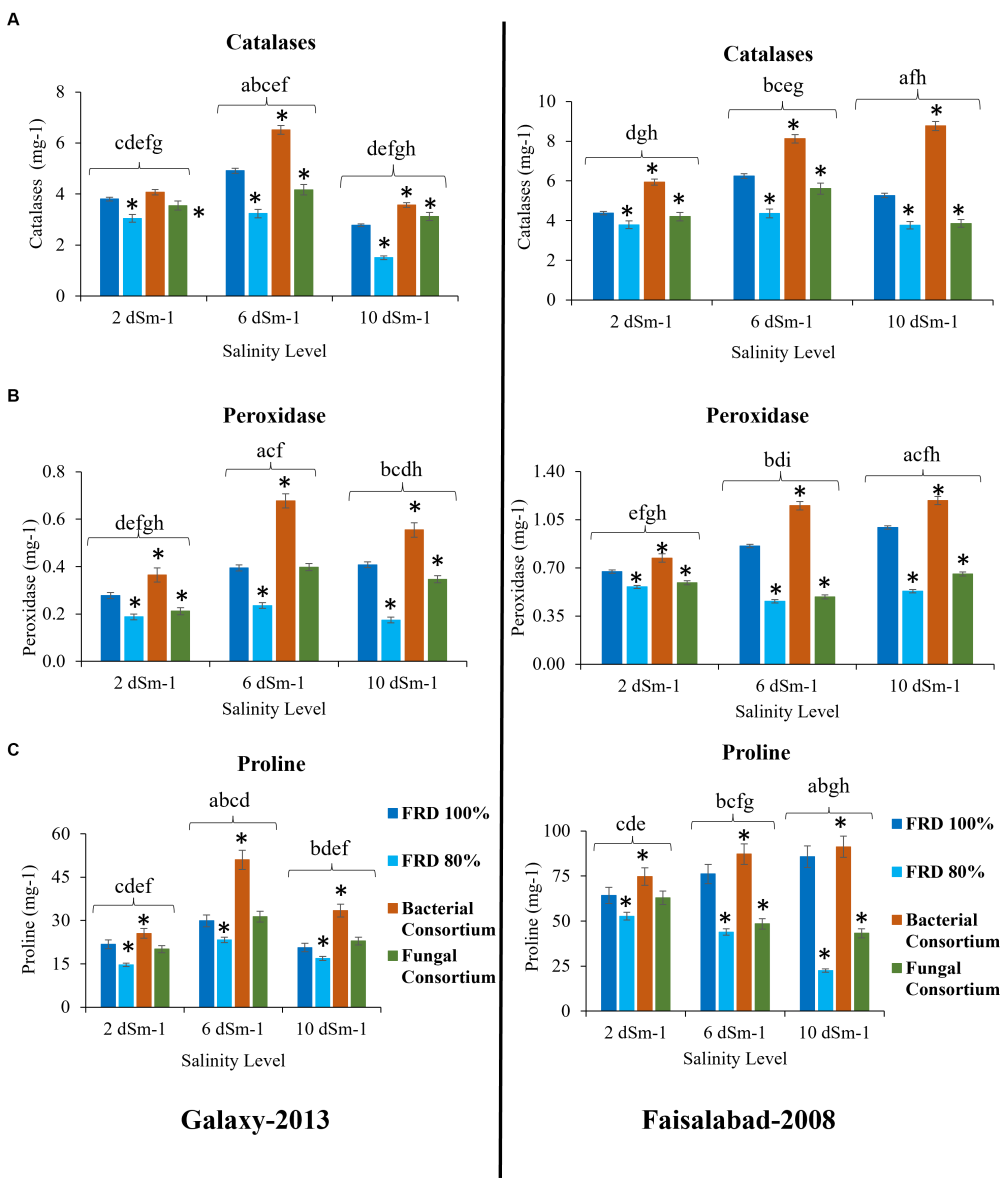
Effect of reduced fertilizer: 80% FRD and bacterial and fungal inoculation on biochemical parameters **(A)** Catalase **(B)** Peroxidase **(C)** free Proline content; at different salinity levels for wheat varieties Galaxy 2013 and Faisalabad 2008 in pot experiment where bars indicate the Mean ± Standard Error of N = 3, n = 9; *p < 0.05, Dunnett’s test relative to control; FRD 100%. Different letters indicate *p* < 0.05, ANOVA followed by Tukey’s HSD test across the test dosage.

For agronomical parameters, in Galaxy 2013, the statistical significance varied with salinity level; however, it was observed that the plants tested with FRD 80% and fungal consortium have shown significantly varied statutes compared to the control and bacterial consortium. During the experiment, it was observed that fungal inoculation improved the agronomical parameters compared to FRD 80% at the vegetative stage. However, no significant effect was observed on plants with respect to agronomical parameters, when studied at maturity levels (Supplementary Data File 1, Tables 5–8). Meanwhile, when plants were subjected to the highest tested NaCl salinity level, i.e., 10 dSm^−1^, a few parameters did exhibit elevated significance for bacterial consortium. A similar trend was observed for Faisalabad 2008, but it was observed that Faisalabad 2008 (Figures 6, 7) was more responsive to tested parameters compared to Galaxy 2013 (Figures 6, 7). A general trend to infer the agronomical statutes is; bacterial consortium ~ FRD 100% > fungal consortium ~ FRD 80% and 2 dSm^−1^ > 6 dSm^−1^ > 10 dSm^−1^, suggesting the low grain yield was directly affected due to the other reducing agronomical parameters with the increase of salinity levels. For metabolic parameters, treating plants with bacterial consortium leads to significantly elevated differences compared to all the other plants for both the varieties. However, with the increase of the NaCl dosage, metabolic parameter decline was observed in all the tested plants. Generally, the significant differences were observed for both varieties at moderate saline conditions (i.e., 6 dSm^−1^) in the following pattern: bacterial consortium > FRD 100% > fungal consortia > FRD 80%, while lower at non-saline conditions (i.e., 2 dSm^−1^) and strongest at high saline conditions (i.e., 10 dSm^−1^) (Figure 8).

### 3.9. Field trials

#### 3.9.1. Soil physicochemical properties

**Figure 9:**
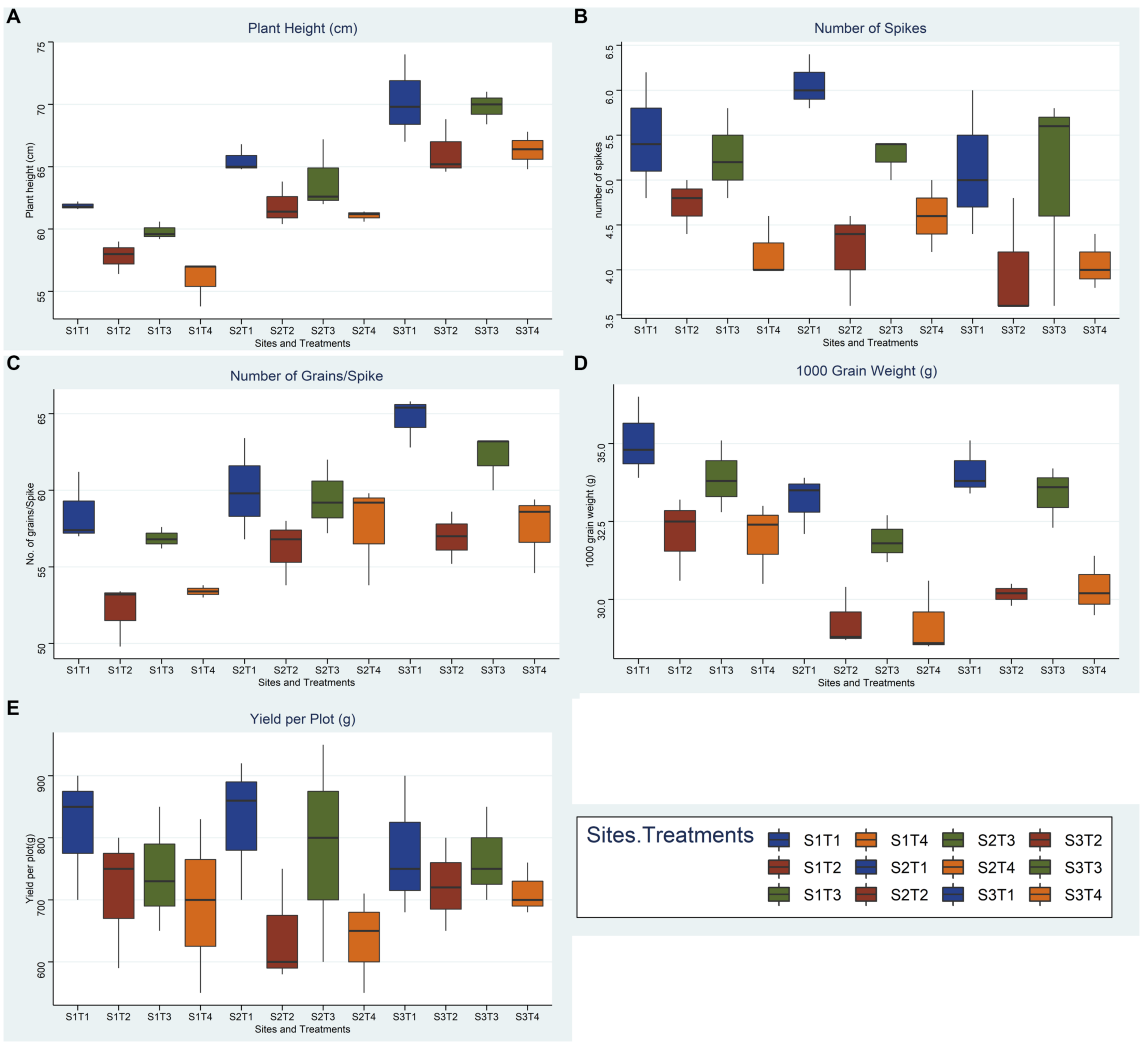
Effect of reduced fertilizer: 80% FRD and bacterial and fungal inoculation compared to 100% FRD, on agronomical parameters **(A)** plant height **(B)** number of spikes **(C)** No. of grains per spike **(D)** 1000 grain weight **(E)** yield per plot; of wheat variety Galaxy 2013 for field trials at three site, where, S1 = Pindi Bhattiyan, S2 = Jhang, S3 = Pakka Anna, T1 = Control with 100% FRD, T2 = Control with 80% FRD, T3 = Bacterial consortium and 80% FRD and T4 = fungal consortium and 80% FRD, where N = 3 and n = 15.

**Figure 10:**
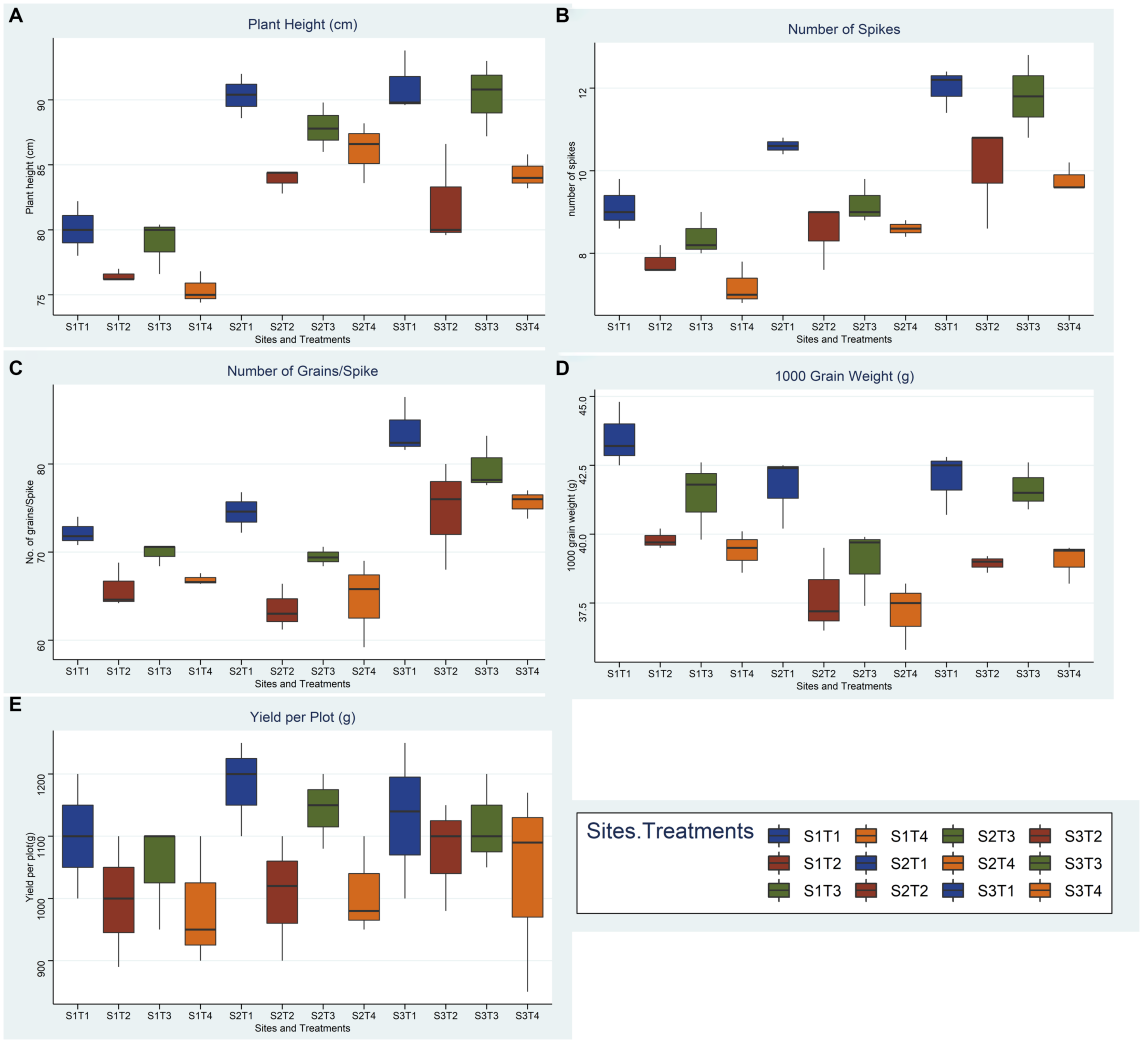
Effect of reduced fertilizer: 80% FRD and bacterial and fungal inoculation compared to 100% FRD, on agronomical parameters **(A)** plant height **(B)** number of spikes **(C)** No. of grains per spike **(D)** 1000 grain weight **(E)** yield per plot; of wheat variety Faisaabad 2008 for field trials at three site, where, S1 = Pindi Bhattiyan, S2 = Jhang, S3 = Pakka Anna, T1 = Control with 100% FRD, T2 = Control with 80% FRD, T3 = Bacterial consortium and 80% FRD and T4 = fungal consortium and 80% FRD, where N = 3 and n = 15.

A summary of soil standard parameters like texture and selected chemical parameters is given in Table 3. Briefly, pHs for soils at three different sites ranging from mildly alkaline for Mouza Sheikhana, Jhang (7.56 ± 0.12) and Pindi Bhattiyan (7.73 ± 0.03) to moderately alkaline for Pakka Anna (8.21 ± 0.09). Soil ECs, at all three different sites, fall under the moderately saline (EC: 4–8) category with Mouza Sheikhana, Jhang having the highest (7.92 ± 0.14) followed by Pakka Anna (7.18 ± 0.09) and Pindi Bhattiyan (6.3 ± 10.27). Typical properties of saline soils (high ionic concentrations and lower OM and N values) were present at these sites. The highest concentration of chlorides (meq L^−1^) was observed for Mouza Sheikhana Jhang (251 ± 4.87) followed by Pakka Anna (247 ± 6) and Pindi Bhattiyan (187 ± 1.69). Water-soluble Na^+^ (g L^−1^) was also highest at Mouza Sheikhana Jhang (31.4 ± 0.97) followed by Pindi Bhattiyan (18 ± 0.28) and Pakka Anna (15.01 ± 4.08). The available P met the recommended normal values for these regions (Figures 9, 10).

**Table 3:** Physicochemical properties of three salt-affected sites selected for field trials.

Location	coordinates	Soil texture	pHs	ECe	Organic matter	Available P	Chlorides	Water soluble K^+^	Water soluble Na^+^	Nitrogen
				(dS m^−1^)	(%)	(mg kg^−1^ soil)	(meq L^−1^)	(g L^−1^)	(g L^−1^)	(%)
SSRI, Pindi Bhattiyan	31°53′45”N, 73°16′34″E	Sandy clay	7.73 ± 0.036	6.31 ± 0.27	1.19 ± 0.117	27.43 ± 1.82	187 ± 1.69	0.43 ± 0.026	18 ± 0.28	0.052 ± 0.004
BSRS II, Pakka Anna, Faisalabad	31°15′00.2”N 72°49′25.0″E	Sandy loam	8.21 ± 0.098	7.18 ± 0.09	0.78 ± 0.098	32.61 ± 0.67	247 ± 6	0.23 ± 0.071	15.01 ± 4.08	0.065 ± 0.007
Farmer’s field, Mouza Sheikhana, Jhang	31°21′52”N, 72°22′31″E	Sandy loam	7.56 ± 0.121	7.92 ± 0.14	1.08 ± 0.21	24.52 ± 2.32	251 ± 4.87	0.29 ± 0.038	31.4 ± 0.97	0.071 ± 0.004

EC: Electrical conductivity; P: Phosphorous; K^+^: Potassium ion and Na^+^: Sodium ion. Values: average ± standard deviation (*n* = 3).

#### 3.9.2. Soil agronomical parameters

Overall, bacterial treatment (T3) performed better compared to fungal treatment (T4) and control with reduced fertilizer (T2) at all three sites, and Faisalabad 2008 showed better outcomes compared to Galaxy 2013 (Figures 9, 10; Supplementary File 1, Tables 9–11). For the studied agronomical parameters, the response to bacterial (T3) and fungal (T4) inoculations in the form of percentage (%) increase as compared to control with 20% reduced fertilizer (T2) at all three sites was calculated. Briefly, a maximum % increase for 1,000 grain weight was observed for bacterial treatment of both varieties, 21.8% for Faisalabad 2008 and 15.9% for Galaxy 2013 at Pakka Anna. Similarly, a maximum increase in plot yield was observed for Faisalabad 2008 at Pakka Anna (10.07%) and Jhang (13.5%), while an increase of 21.7% was observed for Galaxy 2013 at Jhang. Details for all studied agronomical parameters are provided in Supplementary File 1, Table 12.

Statistical analysis using a two-way ANOVA at each location indicated that both treatments and wheat genotypes individually have a significant relationship with almost all parameters considered in this study except yield per plot (Supplementary File 1, Tables 13–15). Combined correlation graphs while considering both wheat varieties separately and all replicates of all treatments collectively at three sites simultaneously showed that the mutual effect of salinity and reduced fertilizer (T2, T3, and T4) is more drastic compared to salinity solely (T1).

Surprisingly, most parameters were significantly or less significantly positively correlated with each other but were, however, also negatively correlated with yield per plot for each variety. For example, Plant height for T1 was positively correlated with flag leaf length (0.775), flag leaf weight (0.779), and number of grains per spike (0.700) for Galaxy 2013 but a negative correlation (−0.395) for 1,000 grain weight and yield per plot (−0.389) was observed. However, for T2, plant height showed positive correlation with number of grains (0.765). Similarly, for T3, plant height showed a positive correlation with flag leaf length (0.847), flag leaf weight (0.0.941), and number of grains per spike (0.781).

**Figure 11:**
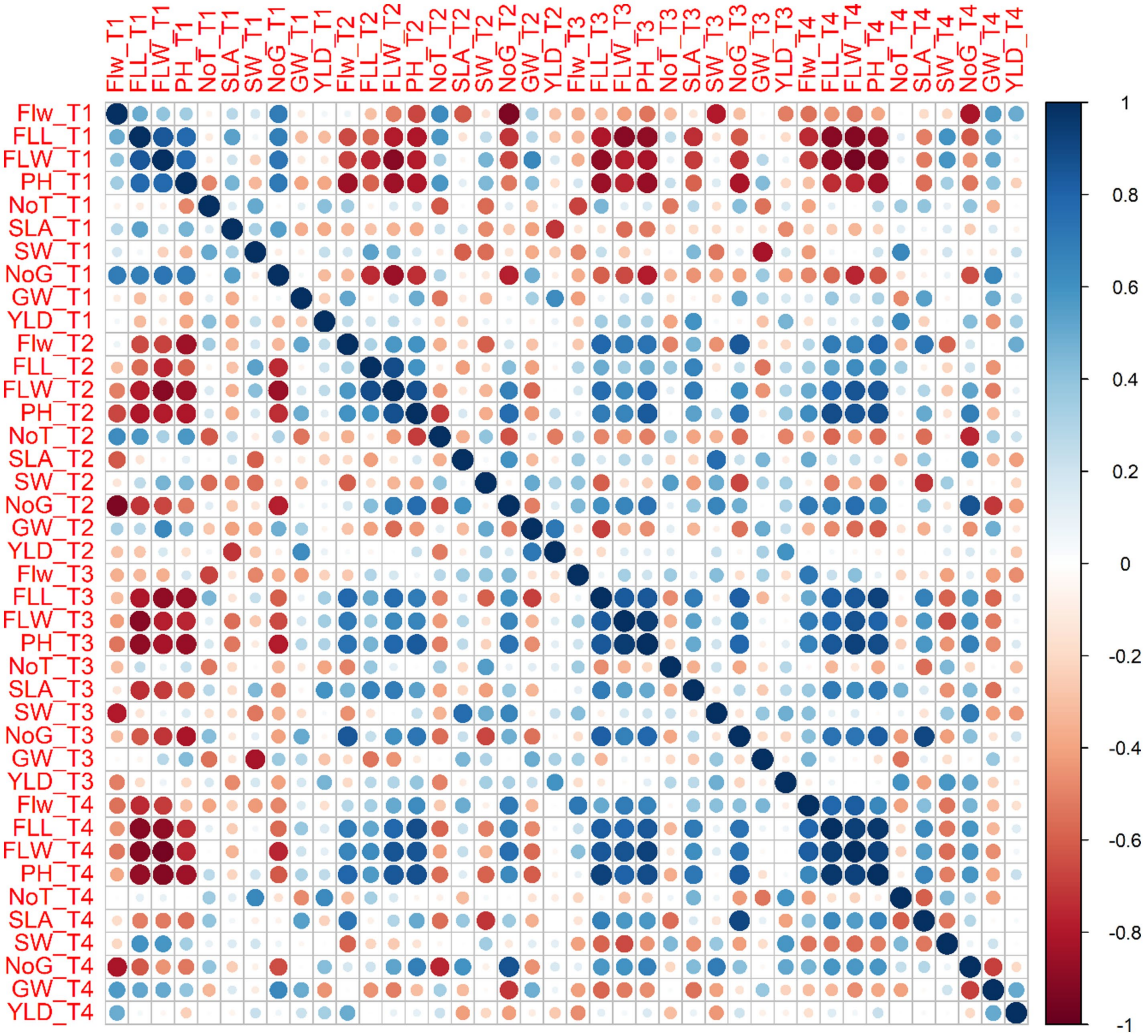
Combined correlation of collective average values for agronomical parameters at three sites for all treatment *viz.* T1 = Control with 100% FRD, T2 = Control with 80% FRD, T3 = Bacterial treatment, and T4 = Fungal treatment for Galaxy 2013 where Flw = Flag leaf width, FLL = flag leaf length, FLW = flag leaf weight, PH = plant height, NoT = Number of tillers, SLA = Spike length with awns, SW=Spike weight, NoG = Number of grains spike^−1^, GW = 1,000 grain weight, and YLD = Yield per plot. The blue and red circles show the positive and negative correlation, respectively. The circle size and the intensity of the color are proportional to the correlation coefficients.

**Figure 12:**
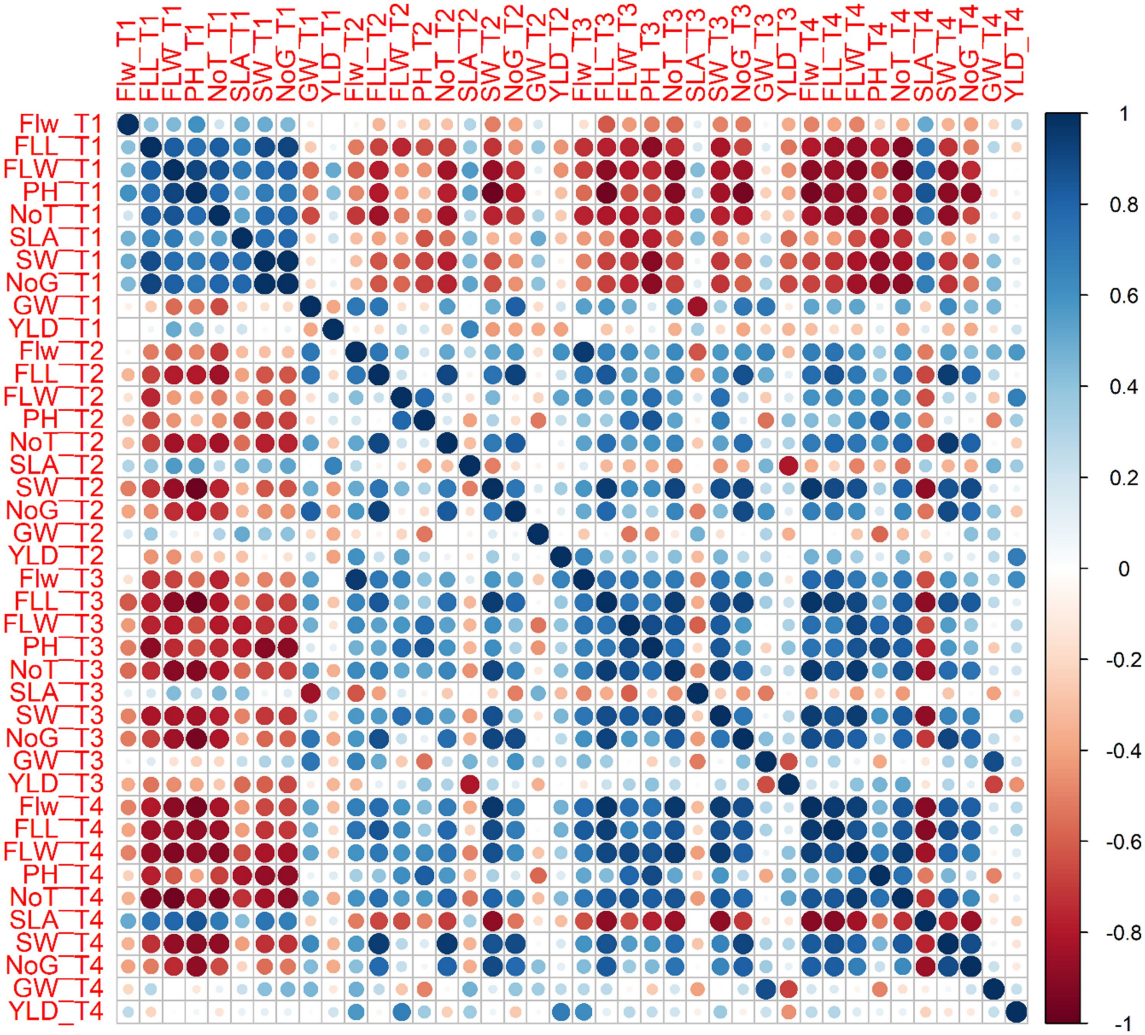
Combined correlation of collective average values for agronomical parameters at three sites for all treatment *viz.* T1 = Control with 100% FRD, T2 = Control with 80% FRD, T3 = Bacterial treatment, T4 = Fungal treatment for Faisalabad 2008. Where, Flw = Flag leaf width, FLL = flag leaf length, FLW = flag leaf weight, PH = plant height, NoT = Number of tillers, SLA = Spike length with awns, SW=Spike weight, NoG = Number of grains spike^−1^, GW = 1,000 grain weight, and YLD = Yield per plot. The blue and red circles showed the positive and negative correlation, respectively. The circles size and the intensity of the color are proportional to the correlation coefficients.

In the case of Faisalabad 2008, in addition to flag leaf length (0.750), flag leaf weight (0.0.910), and number of grains per spike (0.710), plant height was also positively correlated with number of spikes per plant (0.774) for T1. Similarly, yield per plot was only negatively correlated with spike weight (−0.035). For T3. All parameters were positively correlated with plant height except for1000 grain weight (−0.227) and spike length with awns (−0.191). The fungal consortium (T4) showed a higher negative correlation with all parameters (−0.909 to −0.735) for spike length with awns except 1,000 grain weight (−0.094), yield per plot (−0.227), and plant height (−0.519). Details of correlation studies among different agronomical parameters and treatments for each variety are provided in Figures 11, 12.

## 4. Discussion

Despite all the recent advancements and application of the latest technologies for sustainable agriculture, the threat to food security due to continual climate change is still persistent. The expansion of salinity around the globe generally, and in Pakistan particularly, needs special attention (Nawaz A. et al., 2020). Scientists in countries facing the challenges of agricultural lands deterioration due to salinity are giving special emphasis to the exploration of the indigenous microbial flora for the reclamation of saline soils and to exploit the hidden potential of these microbes for crops augmentation in addition to finding the solution through classical and modern approaches like breeding, gene editing, and development of transgenic crops (Perez-Jimenez and Perez-Tornero, 2020; Marghoob et al., 2022). The present study was conducted to explore the indigenous microbial flora of the Indus basin region of Pakistan and its efficacy to overcome the problems associated with low wheat production by mitigating the adverse effect of salinity, and initial promising results indicated the potential of selected strains for salinity stress alleviation in addition to less dependency on chemical fertilizers.

In the current scenario, salt-affected agroecological zones present in the Indus basin region of Pakistan provide an ideal location, as the agricultural sector mainly relies on this region to meet the wheat requirements and needs special attention to achieve the desired yield for the ever-increasing population. Recent but limited culture-dependent and independent studies revealed that relatively less explored indigenous flora might possess multiple PGP traits in addition to being able to withstand hypersaline conditions (Mukhtar et al., 2020; Nawaz A. et al., 2020; Marghoob et al., 2022). Therefore, soil samples were collected from agricultural fields in the Punjab and Sindh provinces from 17 sites (Supplementary File 1, Table 1), where wheat is grown in the winter season and these areas were affected by various levels of salinity. Soil EC values (Supplementary File 1, Table 1) indicated that all these soils fall under the category of saline soils, ranging from moderately saline (4–8) to very strongly saline (>16) according to Abrol et al. (1988). These soil samples were used for the isolation of halotolerant bacteria and their characterization.

Assessment of microbial flora for possession of salt tolerance and PGP traits are a primary prerequisite before their application for salinity stress mitigation in plants (Li et al., 2022). In order to evaluate the MICs for salt tolerance and PGP and other biochemical traits, the 297 initially isolated pure colonies were screened on salt-supplemented media, and only isolates with the capacity to withstand 7% and/ or 10% NaCl concentration (Supplementary File 2, Table 2) were selected for further evaluation. Out of 141 halotolerant selected strains, a majority number showed possession of one or multiple characteristics (Supplementary File 2, Table 3) that can help in plant growth promotion directly or indirectly. Interestingly, only a selected set of isolates was able to produce IAA. This could be explained by a recent study, based on a metagenomic survey of salt-affected soils in the Indus Basin region, that proposed the presence of microbes with a tryptophan-independent indole-3-glycerol phosphate synthase (IGPS) mechanism in these soils and hypothesized that the activity of IGPS might be directly linked with salinity (Marghoob et al., 2022).

On the basis of promising PGP and other biochemical characteristics (Table 1), three bacterial strains, UM 16, UM 58, and UM83, were selected to further investigate their role in stress alleviation and crop improvement. Briefly, all three strains were able to withstand NaCl concentrations up to 10%. All three halotolerant strains were able to solubilize inorganic phosphorous, potassium, and zinc. In addition, these strains have the capability to produce siderophores and EPS and possess amylase enzymes, as these characteristics help microbes to survive and facilitate plant growth in harsh conditions (Batool et al., 2017; Mukhtar et al., 2019). No hemolytic activity was observed on the blood agar medium during initial screening, which indicated their nonpathogenic nature (Payment et al., 1994). 16S rRNA gene sequencing and BLAST results revealed that these strains belong to *Erwinia rhapontici, Pantoea agglomerans*, and *Pantoea* sp., respectively.

The role of PGP strains belonging to the genus *Pantoea* in plant growth improvement has been reported previously, and their ability to display multiple PGP characteristics, including a promising role under biotic and abiotic stresses, is also well established (Mishra et al., 2016; Chakdar et al., 2018; Luziatelli et al., 2020; Lorenzi et al., 2022). Mishra et al. (2016) reported that strains belonging to *Pantoea agglomeran* produce EPS that in addition to helping in osmoregulation under abiotic stresses also improve soil texture through formation of microaggregates and enhance water holding capacity.

Interestingly, similar to many other bacterial families, bacterial strains belonging to the genus *Erwinia* also showcase diverse characteristics from pathogenicity to PGP and stress alleviation (Kang et al., 2014; Saldierna Guzmán et al., 2021; Cochard et al., 2022). Bacterial strains belonging to *E. rhopontici* have been associated with pathogenicity previously; however, a recent study by Kaur et al. (2023) indicated their PGP traits and a consortium containing *E. rhopontici* improved the growth of finger millet even at a reduced dose of chemical fertilizer. The *Erwinia* strain used in this study exhibited no potential threat to the crop as well as to human health. However, further research is recommended to evaluate the pathogenic role of these strains to eliminate the risk of disease on other rotational crops grown in this region.

In addition, the *E. rhopontici* strain used in this study also exhibited ACC deaminase activity. This enzyme is of crucial importance for plant growth in stressed environments including that of salinity as it regulates the excessive production of ethylene by breaking the precursor for ethylene, 1-aminocyclopropane-1-carboxylate (ACC), into ammonia and α-ketobutyrate (Bouffaud et al., 2018; Ben-Laouane et al., 2020).

Studies regarding the understanding of mycorrhizal and fungal communities to improve plant overall health and as biocontrol agents suggested the promising role of these creatures for sustainable agriculture (Faraz et al., 2020; Comite et al., 2021). However, research related to exploring the potential of individual fungal strains as plant growth promoters is limited due to the complicated metabolic mechanisms they possess, making it difficult to gain a conclusive idea of how they are involved in the improved growth of plants. In general, indirect effects on overall plant health and growth parameters are associated with the inoculated fungal strains (Martinez-Medina et al., 2014; Sánchez-Montesinos et al., 2020). Literature survey has shown strains of the genus *Trichoderma* might play a vital role in plant growth and stress alleviation for wheat under abiotic stresses like salinity (Sánchez-Montesinos et al., 2020). Hence, two previously isolated and characterized strains of *Trichoderma*, *T. harzianum*, and *T. viridae* (Faraz et al., 2020) were selected to evaluate the response of wheat crop in the presence of saline conditions.

Similarly, application of microbial strains in the form of a consortium, in comparison to the use of individual strains, for field experiments is considered a more promising approach (Devi et al., 2022). Survival and adaptation chances of microbial strains applied in the form of consortia are higher as compared to individual strains (Sekar et al., 2016; Kaur et al., 2023). However, one strain might hinder the growth of other, and cross streak method is therefore considered a robust and convenient approach to check the compatibility of different strains used in a consortium (Semenov et al., 2007). Additionally, as none of the strains displayed antagonistic activity against each other, the consortia of three selected bacterial strains and both fungal strains were developed separately to examine their role in plant growth under saline conditions.

Salinity may hamper the growth of plants at different stages (Etesami and Maheshwari, 2018; Perez-Jimenez and Perez-Tornero, 2020; Hajiabadi et al., 2021); a set of experiments was therefore used in this study to deeply examine the response of two local wheat cultivars, Faisalabad 2008 and Galaxy 2013 (differed based on salt tolerance), in the presence of these inoculums and at different salinity levels *in vitro* as well as under semi-controlled and field conditions according to protocols described in the literature previously for different crops (Mukhtar et al., 2020; Nawaz A. et al., 2020; Perez-Jimenez and Perez-Tornero, 2020).

Initially, a plate assay for seed germination was performed for both wheat varieties at four different salinity levels (0, 50, 100, and 150 mM NaCl), and results indicated that seed germination was better in the case of Faisalabad 2008 compared to Galaxy 2013. However, the germination rate was inversely proportional to increasing salt concentration. Seeds primed with bacterial inoculum performed better than controls. The results were according to expectations as Faisalabad 2008 is considered somewhat resistant to mild saline conditions while Galaxy 2013 is more susceptible (Kanwal et al., 2011; Nawaz et al., 2013; Ahmad A. et al., 2022), and the literature survey indicated the potential of PGP strains including non-pathogenic *Pantoea* and *Erwinia* spp. (Cochard et al., 2022; Lorenzi et al., 2022) for salinity stress alleviation in crops through different metabolic pathways that confer resistance through direct and indirect mechanisms (Nawaz M. S. et al., 2020; Yang et al., 2020).

Additionally, roots are tissues of the plant more directly exposed to abiotic stresses like salinity compared to other parts (Byrt et al., 2018), and root system architecture studies were therefore an important aspect of understanding the response of roots to salinity in the presence of bioformulation. Different root architecture parameters analyzed in this study (Figures 3–5; Supplementary File 1, Tables 3, 4) indicated that in general, the presence of bioformulations positively correlates with these parameters and helps in suppressing the negative effects of salinity and nutrient deficiency. Interestingly, in both contrasting genotypes, effects of low nutrients and bacterial formulation combinations were surprising at 40 mM for Galaxy 2013 and at 60 mM for Faisalabad 2008, as parameter values deviated from the general trend observed in the study.

Generally, studies related to the comparative effects of salinity or abiotic stress, nutrient variation, and bioformulation on root parameters of wheat are scarce (Rahnama et al., 2011; Robin et al., 2016, 2021). However, limited available data indicate that nutrients and bioformulation affect root growth in a complicated way and a combination of root architecture parameters, biochemical compounds exchange, and the nutrient combination might provide a better understanding of how different parameters affect root structure formation and function of plants when counted collectively (Buwalda et al., 1988; Li et al., 2014; Arif et al., 2019; Guo et al., 2019; de Souza Campos et al., 2021). Also, these parameters were studied in controlled conditions due to limitations of exploring roots architecture from the soil, and as the soil environment is different to the control conditions, studies incorporating root architecture analysis from soil samples are recommended in the future. Advanced technologies like “X-ray computed tomography (CT), Growth and Luminescence Observatory for Roots (GLO-Roots), Soil Rhizotron Phenotyping and computational analysis approach” etc. would facilitate our understanding of the root responses to salinity under natural environmental conditions (Zou et al., 2022).

Concisely, improved grain yield is the ultimate goal of the scientific community, and, based on the available literature, this concept was established that improvement in different agronomical parameters like plant height, number of spikelets, etc., is directly linked with the enhanced grain yield (Tshikunde et al., 2019). However, salinity levels have detrimental effects on different stages of plant growth from seedlings to crop maturity, and they dramatically alter the metabolic pathways (Khaliq et al., 2015; EL Sabagh et al., 2021). On the other hand, seed bio-priming of wheat with PGP microbes improves plant growth and helps increase resistance against biotic and abiotic stresses (Mashabela et al., 2022). To estimate the role of developed PGP fungal and bacterial bioformulations on wheat biochemical and agronomical parameters, pot experiments with three salinity levels and field trials at three different agro-ecological sites were conducted.

Results of the pot experiment indicated that both fungal and bacterial consortia significantly increased the studied biochemical activities (catalase, peroxidase, and free proline content), associated with alleviation of salinity stress, of plants as compared the control at the vegetative stage. Previous studies also showed that the application of bacterial strains helps to induce systemic resistance in plants including wheat against biotic and abiotic stresses through the upregulation of different enzymatic activities especially peroxidase (Khaliq et al., 2015; Byrt et al., 2018; Minaeva et al., 2018). Surprisingly, outcomes of fungal consortium application on overall agronomical parameters and yield at the maturity stage were non-significant. Future studies that investigate the possible reasons are strongly recommended.

Similarly, bacterial inoculation significantly improved the growth and yield of both wheat varieties compared to the control with reduced fertilizer (80% FRD) at all salinity levels and for field trials at three sites. Faisalabad 2008 performed better than Galaxy 2013, which was not surprising as Faisalabad 2008 is considered more salt tolerant compared to Galaxy 2013 (Kanwal et al., 2011; Anwar et al., 2013), and outcomes of the present study further validated its potential for better performance under abiotic conditions, particularly salinity.

However, the combined correlation analysis showed that although the other parameters were significantly correlated with each other, they were negatively or less significantly related to crucial parameters like grain weight and yield per plot for all treatments at three sites in general, which indicates that these genotypes are not suitable for cultivation in saline soils as salinity severely affected these crucial parameters and the response of both genotypes to saline conditions at multiple sites was not uniform. Studying multiple parameters simultaneously is considered an important technique to evaluate genotypes for resistance development against stresses like salinity, as salinity may or may not have an effect on different parameters at different stages, and a genotype with attributes like higher 1,000 grain weight, spike weight, and yield per plot is considered a good candidate for resistant variety (El-Hendawy et al., 2005; Uzair et al., 2022).

Surprisingly, 1,000 grain weight and yield per plot were not positively correlated. A possible explanation for this phenomenon might be the presence of saline patches. Salinity levels changes even within a field in this region and therefore make is difficult to correlate per plot yield with other parameters (Ijaz et al., 2020). However, overall, plants bioprimed with bacterial treatment still performed better compared to the control with 20% reduced fertilizer and fungal treatment.

On the other hand, the presence of salinity already depletes the nutrient availability (Syed et al., 2020) and our use of the reduced dose of chemical fertilizer might also be the reasons for low grain yield and an overall reduction in yield, as nutrient availability is a critical factor for plant overall growth and yield parameters in particular, and a negative correlation to plant height and wheat grain yield is also reported in literature (Islam et al., 2013).

In addition, although the EC levels in T2 (~6dSm^−1^) of the pot experiment were similar to the field conditions of three different sites, differences in multiple agronomical parameters were observed (Supplementary data). This can be explained by the discussion in previous literature where researchers found differences in pot and field trials outcomes and suggested the possible reasons for these obvious differences might be competition for space, light, nutrients, plant interactions with plants, soil, and environmental conditions and these factors have both advantages and disadvantages for overall plant growth (Kawaletz et al., 2014; Poorter et al., 2016; He et al., 2017).

These preliminary investigations indicated that the use of these indigenous microbial florae not only has the potential to augment wheat production under moderately saline conditions but also pave the way to future investigation for a better understanding of plant–microbe interactions under saline conditions. Development and assessment of a mixed consortium of PGP fungal and bacterial strains after further in-depth studies in the future can also be carried out to evaluate their synergistic effect as initial reports of mixed inoculum are promising (Diedhiou et al., 2016; Comite et al., 2021).

## 5. Conclusion

Initial screening and evaluation of halotolerant microbial flora from salt-affected agricultural lands in the Indus basin region indicated the potential of these microbes to enhance wheat production by enhancing multiple growth/agronomical parameters. The outcomes of these preliminary studies would leverage the path for the development of saline-specific bio-fertilizer using indigenous microbial flora to not only combat abiotic stresses like salinity but also to reduce the usage of chemical fertilizers.

## Data availability statement

The datasets presented in this study can be found in online repositories. The names of the repository/repositories and accession number(s) can be found at: https://www.ncbi.nlm.nih.gov/genbank/, OQ318271; https://www.ncbi.nlm.nih.gov/genbank/, OQ318275; https://www.ncbi.nlm.nih.gov/genbank/, OQ318278.

## Author contributions

MM: conceptualization, sampling, experimentation, field trials, data curation, formal analysis, investigation, writing-original draft, and review and editing. AN: sampling, experimentation, field trials, and review and editing. MA: data analysis, writing-original draft, and review and editing. MW and MK: data analysis and review and editing. EI: Rhizo-scanning. MI: sampling, field trials, and review and editing. AI: conceptualization, supervision, and review and editing. FM: conceptualization, funding acquisition, project administration, supervision, validation, and review and editing. All authors contributed to the article and approved the submitted version.

## Funding

We are thankful to the Higher Education Commission (HEC), Pakistan, for a grant under the title of HEC-NRPU project #8508, “Development of saline soil specific Biofertilizer.”

## Conflict of interest

The authors declare that the research was conducted in the absence of any commercial or financial relationships that could be construed as a potential conflict of interest.

## Publisher’s note

All claims expressed in this article are solely those of the authors and do not necessarily represent those of their affiliated organizations, or those of the publisher, the editors and the reviewers. Any product that may be evaluated in this article, or claim that may be made by its manufacturer, is not guaranteed or endorsed by the publisher.
