# Supplementary material for: Assessment of halotolerant bacterial and fungal consortia for augmentation of wheat in saline soils
Source: Front Microbiol. 2023 Jun 30;14:1207784. doi: 10.3389/fmicb.2023.1207784 (PMC10347533; doi:10.3389/fmicb.2023.1207784)
Supplement: Supplementary file 1 [file Data_Sheet_1.PDF]

Supplementary Table 1: Basic details of sample collection sites (Location coordinates, pHs and electrical conductivity (EC))

Sr. No.	Sampling site	Latitude	Longtitude	EC (dSm^{-1})	pH
**1**	Khewra Road	32.6134	73.0239	7.18±0.34	8.53± 0.09
**2**	Choa Saidan Shah-Kallar Kahar Road	30.4319	71.9958	6.08±0.56	7.52±0.011
**3**	Choa Saidan Shah-Kallar Kahar Road	32.7664	72.7341	6.76±0.94	7.68±0.025
**4**	Choa Saidan Shah-Kallar Kahar Road	32.7655	72.738	9.67±1.21	7.12±0.017
**5**	Choa Saidan Shah-Kallar Kahar Road	32.7485	72.7574	8.34±0.67	9.02±0.082
**6**	Talagang-Kallar Kahar Road	32.7863	72.6968	7.79±0.55	7.23±0.041
**7**	Talagang-Kallar Kahar Road	32.7885	72.6823	15.66±0.45	8.41±0.032
**8**	Lillah Road	32.5791	72.8075	8.97±0.25	7.82±0.670
**9**	Lillah Road	32.5851	72.8931	24.56±1.32	7.81±0.091
**10**	Lillah Road	32.5936	73.027	18..33±0.97	7.53±0.027
**11**	Ghubrika, Pindi Bhattiyan	31.8173	73.20805	10.66±1.08	8.65±0.018
**12**	Burjian Wala, Chiniot	31.8055	73.1547	15.38±0.54	8.32±0.46
**13**	Toba Tek Singh	30.9558	72.4766	31.55±1.76	7.61±0.061
**14**	Doluwala	31.8026	73.1768	8.76±0.69	7.45±0.013
**15**	Pakka Anna	31.2455	72.7981	17.16±1.16	8.58±0.036
**16**	Dad Leghari, Ghotki	27.9474	69.6663	31.98±0.35	8.06±0.132
**17**	Bago Bhutto, Daharki	28.0403	69.6561	35.34±1.02	7.61±0.05

Values: Mean±Standard deviation (n=3)

Supplementary Table 2: Primer and PCR profile details for amplification of 16S rRNA gene of bacteria.

Region of interest	Primers	Sequences	Initial step	Denaturation	Annealing	Elongation	Cycles	Final step	Reference
**16S rRNA of Bacteria**	fD1<br><br>rD1	5’-AGAGTTTGATCCTGGCTCAG-3’<br><br>5’-AAGGAGGTGSTCCAGCC-3’	95 °C, 5 min	94 °C, 60 sec	52.5 °C, 60 sec	72 °C, 60 sec	32	72 °C, 10 min	(Besnson et al., 1996)

Supplementary Table 3: Effects of different concentration of Hoagland solution (C100;100% and C80; 80%) and Bacterial treatment (BT; bacterial consortium and 80% Hoagland solution) on selected root structure traits of wheat variety Faisalabad 2008 at different NaCl concentrations (0, 20,40, 60 and 80 mM).

NaCl Conc. (mM)	Treatment	Length (cm)	Surface Area (cm^2)	Average Diameter (mm)	Root Volume (cm^3)	Tips
**0**	100%	64.76±2.32	11.64±0.76	0.33±0.01	0.094±0.002	192.67±1.52
	80%	51.36±1.47	10.42±0.57	0.35±0.02	0.087±0.003	158.66±2.08
	Bact. Consortium	93.87±1.23	13.09±0.51	0.33±0.003	0.107±0.002	165.33±2.51
**20**	100%	105.55±2.78	15.14±0.77	0.33±0.01	0.110±0.001	211.33±2.08
	80%	96.99±1.64	13.65±0.81	0.31±0.001	0.107±0.001	157.67±2.081
	Bact. Consortium	122.44±2.77	17.85±0.18	0.44±0.009	0.116±0.001	205±3.60
**40**	100%	124.80±2.51	10.60±0.45	0.39±0.005	0.083±0.006	154.67±4.041
	80%	113.78±1.86	8.96±0.89	0.31±0.008	0.078±0.004	122.33±2.51
	Bact. Consortium	152.48±3.06	12.11±0.29	0.37±0.01	0.095±0.004	148.33±2.51
**60**	100%	92.70±3.52	9.37±0.55	0.35±0.002	0.096±0.0005	196±2.64
	80%	71.42±1.16	7.79±0.54	0.32±0.01	0.084±0.005	165.33±3.05
	Bact. Consortium	85.46±2.48	11.64±1.41	0.34±0.01	0.095±0.001	182.67±2.51
**80**	100%	102.61±3.03	8.19±0.22	0.25±0.004	0.061±0.001	212.67±3.05
	80%	81.37±1.64	6.61±0.45	0.20±0.008	0.043±0.002	93. 7±2.08
	Bact. Consortium	95.47±1.94	7.81±0.32	0.28±0.01	0.056±0.001	196.33±2.51

Mean values ± Standard deviation where n=3

Supplementary Table 4: Effects of different concentration of Hoagland solution and Bacterial treatment on selected root structure traits of wheat variety Galaxy 2013 at different NaCl concentrations.

NaCl Conc. (mM)	Treatment	Length (cm)	Surface Area (cm^2)	Average Diameter (mm)	Root Volume (cm^3)	Tips
**0**	100%	87.45±0.60	9.73±0.28	0.34±0.006	0.082±0.002	264±3.60
	80%	82.26±1.76	10.01±0.12	0.32±0.005	0.070±0.001	198.33±3.21
	Bact. Consortium	122.33±1.10	12.04±0.89	0.36±0.003	0.094±0.002	255.66±4.16
**20**	100%	102.92±2.11	9.95±0.14	0.31±0.004	0.086±0.0005	266.66±4.72
	80%	80.06±4.31	5.97±0.25	0.30±0.015	0.065±0.001	193.33±5.68
	Bact. Consortium	139.44±2.11	14.30±0.54	0.33±0.001	0.121±0.001	250±4.58
**40**	100%	144.86±2.24	15.32±1.48	0.34±0.008	0.141±0.003	286±3.60
	80%	137.19±1.24	13.45±1.32	0.32±0.004	0.113±0.005	281±5.29
	Bact. Consortium	176.02±2.48	15.91±0.62	0.29±0.004	0.129±0.0005	334.33±3.05
**60**	100%	90.71±1.19	8.67±0.46	0.34±0.002	0.069±0.001	153.66±2.51
	80%	63.41±1.03	7.40±0.12	0.32±0.004	0.066±0.0005	134.66±2.08
	Bact. Consortium	75.50±1.38	7.63±0.19	0.34±0.006	0.063±0.001	147.66±2.08
**80**	100%	96.29±1.29	10.22±0.17	0.31±0.003	0.065±0.001	128.33±3.05
	80%	88.89±1.65	9.49±0.23	0.26±0.006	0.054±0.001	84.33±4.16
	Bact. Consortium	96.98±2.05	10.44±0.41	0.33±0.004	0.087±0.002	119.66±4.16

Where; C100=100% Hoagland solution and C80= 80% Hoagland solution, BT= bacterial consortium and 80% Hoagland solution and 0, 20,40, 60 and 80 are NaCl concentrations in mM. **V**alues; Mean ± Standard deviation where n=3

Supplementary table 5: Effects of bacterial and fungal consortium on selected biochemical parameters of two wheat varieties at three different salt concentrations in a pot experiment.

EC	Treatment	Catalase (Units mg^{-1} protein)		Peroxidase (Units mg^{-1} protein)		Proline (µmol g^{-1} f.wt.)	
		Gxy-13	Fsd-08	Gxy-13	Fsd-08	Gxy-13	Fsd-08
2 dSm^{-1}	T1	3.8±0.23	4.37±0.14	0.27±0.019	0.67±0.023	21.77±1.39	64.22±2.27
	T2	3.04±0.17	3.79±0.15	0.18±0.013	0.56±0.021	14.67±1.32	52.66±2
	T3	4.07±0.33	5.93±0.16	0.36±0.016	0.77±0.020	25.56±2.06	74.66±2.12
	T4	3.54±0.26	4.2±0.18	0.21±0.013	0.59±0.019	20.11±1.61	62.88±1.83
6 dSm^{-1}	T1	4.91±0.36	6.24±0.21	0.39±0.019	0.85±0.017	29.88±1.16	76.11±1.76
	T2	3.23±0.15	4.36±0.17	0.23±0.016	0.45±0.020	23.33±1.41	43.88±2.57
	T3	6.51±0.24	8.12±0.19	0.67±0.017	1.15±0.034	51±2.82	87.11±2.47
	T4	4.17±0.25	5.61±0.17	0.39±0.021	0.48±0.022	31.33±3.39	48.44±1.58
10 dSm^{-1}	T1	2.77±0.21	5.27±0.14	0.40±0.019	0.99±0.029	20.66±3.08	85.66±2.34
	T2	1.5±0.24	3.76±0.20	0.17±0.015	0.53±0.019	16.89±2.93	22.44±1.74
	T3	3.56±0.33	8.76±0.21	0.55±0.022	1.18±0.022	33.44±2.65	91.22±2.43
	T4	3.12±0.17	3.85±0.22	0.27±0.019	0.65±0.030	22.88±2.36	43.22±2.43

Where; T1= 100% FRD, T2= 80% FRD, T3= bacterial consortium and 80% FRD, T4= fungal consortium and 80% FRD. wheat varieties; Gxy-13= Galaxy 2013; Fsd-08= Faisalabad 2008. F.wt= fresh weight of leave. Values: average ± standard deviation where n= (3 plants each from 3 pots) =9

Supplementary table 6: Effects of bacterial and fungal consortium on selected agronomical parameters of two contrasting wheat varieties at three different salt concentrations in a pot experiment.

EC (dSm^{-1})	Treatment	Shoot length (cm)		Shoot dry weight (g)		Number of spikes		Length of spikes (cm)		100 grain weight (g)	
		**Fsd-08**	**Gxy-13**	**Fsd-08**	**Gxy-13**	**Fsd-08**	**Gxy-13**	**Fsd-08**	**Gxy-13**	**Fsd-08**	**Gxy-13**
**2**	**T1**	96.78±4.6	69.44±5.43	1.34±0.09	1.11±0.15	14.77±1.71	9.22±0.6	14.89±1.17	8.21±0.5	4.26±0.07	3.87±0.17
	**T2**	75.55±3.4	64.55±5.68	0.99±0.08	0.95±0.06	10.33±1.32	8.33±0.8	13.11±1.27	7.64±0.5	3.9±0.11	3.46±0.10
	**T3**	95.44±4.1	69.22±4.05	1.11±0.12	1.07±0.11	16.33±1.41	8.89±0.6	15.22±1.20	8.48±0.4	4.25±0.1	3.91±0.09
	**T4**	81±4.38	63.44±4.90	0.99±0.06	0.95±0.06	11.88±1.36	8.33±0.7	13.33±1.58	7.76±0.6	3.95±0.08	3.47±0.08
**6**	**T1**	90.22±3.3	58.56±3.04	0.94±0.03	0.91±0.05	10.67±1.5	9.11±0.6	13.22±1.30	8.16±0.4	3.22±0.1	2.85±0.50
	**T2**	69±2.91	54.78±3.96	0.86±0.02	0.80±0.07	8.33±1.32	8.33±0.8	10.67±0.86	7.62±0.4	2.93±0.1	2.32±0.05
	**T3**	92.44±2.4	60.33±3.74	0.96±0.02	0.93±0.04	11.89±1.27	9.11±0.6	11.33±0.86	8.64±0.3	3.1±0.1	2.92±0.49
	**T4**	70.22±2.3	55.67±3.53	0.91±0.03	0.77±0.06	7.77±1.30	8.11±0.7	10±0.70	7.5±0.52	2.89±0.09	2.37±0.11
**10**	**T1**	58±2.23	44±2.95	0.93±0.01	0.6±0.04	4.55±1.13	4.33±0.8	12±1.41	6.71±0.2	1.72±0.1	1.54±0.18
	**T2**	52±1.93	38.56±3.71	0.84±0.02	0.52±0.03	3±0.70	4±1.11	9.22±0.67	6.36±0.1	1.09±0.09	1.08±0.15
	**T3**	61.89±1.5	42.89±2.67	0.97±0.02	0.54±0.03	4.88±1.05	4.33±0.8	11.33±0.86	6.71±0.2	1.31±0.1	1.35±0.15
	**T4**	54.55±1.7	39.55±2.50	0.87±0.02	0.51±0.02	3.22±0.97	4±0.70	9.33±0.70	6.3±0.24	1.12±0.1	1.07±0.12

Where; T1: 100% FRD, T2: 80% FRD, T3: bacterial consortium and 80% FRD, T4: fungal consortium and 80% FRD, wheat varieties; Gxy-13: Galaxy 2013; Fsd-08: Faisalabad 2008. Values: average ± standard deviation where n= (3 plants each from 3 pots) =9

Supplementary Table 7: Increase (%age) in different agronomical parameters of wheat varieties, Galaxy 2013 and Faisalabad 2008, with bacterial and fungal inoculations as compared to control with reduced fertilizer (80% FRD) at three different salt concentrations in a pot experiment.

Salinity level	Treatment	SL		SDW		No. of Spike		length of spike (cm)		100 grain weight (g)	
		Gxy 13	Fsd 08	Gxy 13	Fsd 08	Gxy 13	Fsd 08	Gxy 13	Fsd 08	Gxy 13	Fsd 08
$2dsm^{-1}$	BT	7.229	26.324	12.865	12.192	6.667	58.065	10.901	16.102	12.981	9.117
	FT	-1.721	7.206	0.585	0.447	0.000	15.054	1.453	1.695	0.321	1.425
$6dsm^{-1}$	BT	10.142	33.977	16.231	10.997	9.333	42.667	13.411	6.250	25.837	5.682
	FT	1.623	1.771	-4.402	4.476	-2.667	-6.667	-1.603	-6.250	2.057	-1.515
$10dsm^{-1}$	BT	11.239	19.017	3.390	15.334	8.333	62.963	5.594	22.892	24.490	20.408
	FT	2.594	4.915	-1.695	2.228	0.000	7.407	-0.874	1.205	-2.041	3.061

Where; Gxy 13= Galaxy 2013, Fsd 08= Faisalabad 2008, BT= Bacterial Treatment, FT= Fungal Treatment, SL= Shoot Length, SDW= Shoot Dry Weight

Supplementary Table 8: Increase (%age) in different biochemical parameters of wheat varieties, Galaxy 2013 and Faisalabad 2008, with bacterial and fungal inoculations as compared to control with reduced fertilizer (80% FRD) at three different salt concentrations in a pot experiment.

Variety	Salinity level	Treatment	CAT (units mg^{-1})		POD (units mg^{-1})		Proline (µmol g^{-1})	
			Gxy 13	Fsd 08	Gxy 13	Fsd 08	Gxy 13	Fsd 08
Galaxy 13	$2dsm^{-1}$	BT	33.577	56.598	94.083	37.426	74.242	41.772
		FT	16.423	10.850	13.018	5.545	37.121	19.409
	$6dsm^{-1}$	BT	101.375	86.480	187.736	152.068	118.571	98.481
		FT	28.866	28.827	68.868	7.056	34.286	10.380
	$10dsm^{-1}$	BT	137.778	132.743	217.834	124.319	98.026	306.436
		FT	108.148	2.360	98.726	23.690	35.526	92.574

Where; BT= Gxy 13= Galaxy 2013, Fsd 08= Faisalabad 2008, Bacterial Treatment, FT= Fungal Treatment, CAT= Catalase, POD= Peroxidase,

Supplementary Table 9: Effect of different treatments on agronomical parameters of wheat varieties Galaxy 2013 and Faisalabad 2008 for field trials conducted at Pindi Bhattiyan.

		Days of flowering	Days of maturity	Flag leaf weight (g)	Flag leaf length (cm)	Flag leaf width (cm)	Plant height (cm)	Number of spikes/ plants	Spike length with awns (cm)	Spike weight (g)	Number of grains/ spikes	1000 grains weight (g)	Plot yield (Kg)
**Galaxy 2013**	T1	118±1	155±3	0.262±0.125	13±1.81	0.94±0.15	61.87±5.63	5.46±1.68	9.2±1.86	2.53±0.24	58.53±7.28	35.06±1.32	816.67±104.08
	T2	120±2	159±3	0.186±0.052	9.93±1.48	0.78±0.15	57.8±5.94	4.73±1.53	8.4±1.64	2.34±0.27	52.13±5.55	32.1±1.34	713.33±109.69
	T3	119±2	155±2	0.278±0.089	11.67±1.72	0.9±0.16	59.8±4.96	5.27±1.28	8.73±1.53	2.46±0.24	56.87±5.41	33.9±1.15	743.33±100.66
	T4	120±2	158±3	0.206±0.051	9.67±1.76	0.81±0.14	55.93±5.51	4.2±1.56	8.47±1.30	2.31±0.20	53.4±5.92	31.96±1.30	693.33±140.12
**Faisal-abad 2008**	T1	114±1	147±2	0.359±0.048	23.67±4.49	1.18±0.16	80.07±5.65	9.13±1.45	12±1.36	3.19±0.34	72.2±5.24	43.5±1.179	1100±100
	T2	118±2	150±1	0.26±0.058	21.2±2.95	0.89±0.13	76.46±4.79	7.8±1.89	10.2±1.32	2.89±0.31	65.86±4.34	39.8±0.36	996.67±105.04
	T3	115±2	147±1	0.315±0.084	23.13±3.96	1.02±0.17	79±5.65	8.4±1.68	11.53±1.55	3.02±0.29	69.87±4.59	41.4±1.44	1050±86.60
	T4	117±3	149±2	0.251±0.046	20.6±2.89	0.87±0.20	75.4±4.83	7.2±1.61	10.33±1.34	2.92±0.29	66.86±4.14	39.4±0.75	983.33±104.08

Values: Mean± Standard deviation n= (3*5) =15 where; T1= FRD 100%, T2 = FRD 80%, T3= Bacterial consortium, T4= Fungal consortium

Supplementary Table 10: Effect of different treatments on agronomical parameters of wheat varieties Galaxy 2013 and Faisalabad 2008 for field trials conducted at Jhang.

		Days of flowering	Days of maturity	Flag leaf weight (g)	Flag leaf length (cm)	Flag leaf width (cm)	Plant height (cm)	Number of spikes/ plants	Spike length with awns (cm)	Spike weight (g)	Number of grains /spikes	1000 grains weight (g)	Plot yield (g)
**Galaxy 2013**	T1	118±1	155±3	0.26±0.09	14.33±1.58	1.07±0.11	65.53±4.98	6.07±1.83	10.2±1.78	2.66±0.27	60±6.58	33.17±0.94	826.67±113.72
	T2	120±2	159±3	0.19±0.04	11±1.36	0.9±0.13	61.87±5.35	4.2±1.93	8.73±1.83	2.25±0.32	56.2±6.52	29.3±0.95	643.33±92.91
	T3	119±2	155±2	0.28±0.06	13.47±1.55	0.96±0.15	63.93±4.97	5.26±2.05	9.26±1.67	2.50±0.39	59.47±6.76	31.9±0.75	783.33±175.6
	T4	120±2	158±3	0.21±0.05	10.53±1.68	0.92±0.15	61.06±5.27	4.6±1.72	8.73±1.94	2.32±0.33	57.6±7.61	29.23±1.18	636.67±80.83
**Faisalabad 2008**	T1	114±1	147±2	0.37±0.05	26.33±3.53	1.41±0.18	90.33±4.79	10.6±1.63	11.93±1.87	3.33±0.36	74.53±6.82	41.7±1.3	1183.33±76.4
	T2	118±2	150±1	0.27±0.06	21.8±2.76	1.03±0.13	83.86±4.40	8.53±1.68	10±1.30	2.91±0.33	63.53±10.92	37.73±1.57	1006.67±100.66
	T3	115±2	147±1	0.33±0.08	23.93±3.78	1.22±0.17	87.86±4.59	9.2±1.82	11.4±1.72	3.27±0.34	69.47±8.50	39±1.39	1143.33±60.3
	T4	117±3	149±2	0.26±0.05	22±2.97	1.02±0.12	86.13±4.83	8.6±1.55	10.13±1.35	3.01±0.25	64.67±10.31	37.16±1.23	1010±79.37

Values: Mean±Standard deviation n=(3*5)=15 where; T1= FRD 100%, T2 = FRD 80%, T3= Bacterial consortium, T4= Fungal consortium

Supplementary Table 11: Effect of different treatments on agronomical parameters of wheat varieties Galaxy 2013 and Faisalabad 2008 for field trials conducted at Pakka Anna.

		Days of flowering	Days of maturity	Flag leaf weight (g)	Flag leaf length (cm)	Flag leaf width (cm)	Plant height (cm)	Number of spikes/ plants	Spike length with awns (cm)	Spike weight (g)	Number of grains/ spikes	1000 grains weight (g)	Plot yield (g)
**Galaxy 2013**	T1	118±1	155±3	0.30±0.06	15±2.85	1.23±0.19	70.26±7.07	5.13±2.61	9.8±2.62	2.48±0.59	64.66±7.0	34.1±0.88	776.66±112.39
	T2	120±2	159±3	0.24±0.04	11.33±2.32	0.96±0.22	66.2±6.52	4±2.33	8.6±2.35	2.24±0.57	56.93±7.14	30.16±0.35	723.33±75.05
	T3	119±2	155±2	0.29±0.07	14.27±3.10	1.11±0.24	69.8±7.62	5±3.07	9.53±2.13	2.47±0.52	62.13±6.49	33.36±0.97	766.6±76.4
	T4	120±2	158±3	0.24±0.05	12.06±3.15	1.06±0.21	66.33±7.24	4.07±2.43	9.26±2.05	2.20±0.42	57.53±8.87	30.36±0.96	713.3±41.6
**Faisal-abad 2008**	T1	114±1	147±2	0.37±0.07	31±5.84	1.54±0.34	91.06±9.26	12±2.45	12.93±2.01	3.85±0.93	83.86±9.41	42±1.13	1130±125.3
	T2	118±2	150±1	0.31±0.06	28.6±5.75	1.06±0.22	82.06±8.20	10.07±2.73	9.93±2.28	3.52±0.79	74.66±8.80	38.93±0.30	1076.67±87.36
	T3	115±2	147±1	0.35±0.07	31.8±5.69	1.3±0.39	90.33±8.72	11.8±2.80	11.13±1.99	3.71±0.75	79.66±10.34	41.67±0.86	1116.66±76.37
	T4	117±3	149±2	0.32±0.05	28.2±4.23	1.21±0.24	84.33±8.89	9.8±1.93	9.26±2.25	3.53±0.64	75.6±7.72	39.03±0.72	1036.7±166.5

Values: Mean± Standard deviation n= (3*5)=15 where; T1= FRD 100%, T2 = FRD 80%, T3= Bacterial consortium, T4= Fungal consortium

Supplementary Table 12: Increase (%age) in different agronomical parameters of contrasting wheat varieties with bacterial and fungal inoculation as compared to control with reduced fertilizer (80% FRD) for field trials conducted at Pindi Bhattiyan, Jhang and Pakka Anna.

VR	TR	S	Flag leaf weight (g)	Flag leaf length (cm)	Flag leaf width (cm)	Plant Height (cm)	Number of spikes/ plants	Spikes length with awns (cm)	Spike weight (g)	Number of grains/ spikes	1000 grains weight (g)	Plot yield (g)
**Galaxy 2013**	**BT**	**Pindi Bhattiyan**	49.821	17.450	15.385	3.460	11.268	3.968	5.088	9.079	5.607	4.206
	**FT**		11.111	-2.685	4.274	-3.230	-11.268	0.794	-1.592	2.430	-0.415	-2.804
**Faisalabad 2008**	**BT**		21.282	9.119	14.925	3.313	7.692	13.072	4.094	6.073	4.020	5.351
	**FT**		-3.590	-2.830	-2.239	-1.395	-7.692	1.307	0.828	1.518	-1.005	-1.338
**Galaxy 2013**	**BT**	**Jhang**	43.003	22.424	7.407	3.341	25.397	6.107	11.160	5.813	8.874	21.762
	**FT**		8.532	-4.242	2.222	-1.293	9.524	0.000	3.197	2.491	-0.228	-1.036
**Faisalabad 2008**	**BT**		23.000	9.786	18.065	4.769	7.812	14.000	12.724	9.339	3.357	13.576
	**FT**		-2.250	0.917	-0.645	2.703	0.781	1.333	3.691	1.784	-1.502	0.331
**Galaxy 2013**	**BT**	**Pakka Anna**	19.945	25.882	15.972	5.438	25.000	10.853	19.945	25.882	15.972	5.438
	**FT**		1.939	6.471	10.417	0.201	1.667	7.752	1.939	6.471	10.417	0.201
**Faisalabad 2008**	**BT**		13.578	11.189	21.875	10.073	17.219	12.081	13.578	11.189	21.875	10.073
	**FT**		2.586	-1.399	13.750	2.762	-2.649	-6.711	2.586	-1.399	13.750	2.762

Where; VR= wheat variety, TR= treatment, S= Site, BT= Bacterial treatment, FT= Fungal treatment

Supplementary Table 13: Comparison of contrasting wheat genotypes and treatments on plant agronomical attributes in field trials conducted at Jhang, using two-way ANOVA.

Traits	Treatment	Genotype	Treatment: Genotype
**Plant Height (cm)**	***	***	NS
**Flag Leaf Width**	***	***	**
**Flag Leaf length (cm)**	***	***	NS
**Flag Leaf weight (g)**	***	***	*
**Number of Tillers**	***	***	NS
**Spike Length with Awns (cm)**	***	***	NS
**Spike Weight (g)**	***	***	NS
**Number of Grains Spike^{-1}**	**	***	NS
**1000 Grain Weight (g)**	***	***	NS
**Yeild per Plot**	*	***	NS

Significance codes: 0 '***' 0.001 '**' 0.01 '*' 0.05 '.' 0.1 'NS (non-significant)' 1

Supplementary Table 14: Comparison of contrasting wheat genotypes and treatments on plant agronomical attributes in field trials conducted at Pindi Bhattiyan, using two-way ANOVA.

Traits	Treatment	Genotype	Treatment: Genotype
**Plant Height (cm)**	***	***	NS
**Flag Leaf Width**	***	***	NS
**Flag Leaf length (cm)**	***	***	NS
**Flag Leaf weight (g)**	***	***	NS
**Number of Tillers**	***	***	NS
**Spike Length with Awns (cm)**	***	***	NS
**Spike Weight (g)**	***	***	NS
**Number of Grains Spike^{-1}**	***	***	NS
**1000 Grain Weight (g)**	***	***	NS
**Yeild per Plot**	NS	***	NS

Significance codes: 0 '***' 0.001 '**' 0.01 '*' 0.05 '.' 0.1 'NS (non-significant)' 1

Supplementary Table 15 : Comparison of contrasting wheat genotypes and treatments on plant agronomical attributes in field trials conducted at Pakka Anna, using two-way ANOVA.

Traits	Treatment	Genotype	Treatment: Genotype
**Plant Height (cm)**	***	***	NS
**Flag Leaf Width**	***	***	NS
**Flag Leaf length (cm)**	**	***	NS
**Flag Leaf weight (g)**	***	***	NS
**Number of Tillers**	**	***	NS
**Spike Length with Awns (cm)**	***	***	**
**Spike Weight (g)**	*	***	NS
**Number of Grains Spike^{-1}**	***	***	NS
**1000 Grain Weight (g)**	***	***	NS
**Yeild per Plot**	NS	***	NS

Significance codes: 0 ‘***’ 0.001 ‘**’ 0.01 ‘*’ 0.05 ‘.’ 0.1 ‘NS (non-significant)’ 1
