# Supplementary material for: Assessment of halotolerant bacterial and fungal consortia for augmentation of wheat in saline soils
Source: Front Microbiol. 2023 Jun 30;14:1207784. doi: 10.3389/fmicb.2023.1207784 (PMC10347533; doi:10.3389/fmicb.2023.1207784)
Supplement: Supplementary file 2 [file Data_Sheet_2.pdf]

Supplementary Table 1: Isolation and purification of halotolerant bacterial strains from 17 sampling sites

Sr. No.	Isolates Initial Codes	Sampling site	Coordinates		Dilution factor	LB media	LB supplemented with 1% NaCl
			Latitude	Longitude			
1	KR1	Khewra Road	32.6134	73.0239	1×10^{-3}	✓	✓
2	KR2	Khewra Road	32.6134	73.0239	1×10^{-3}	✓	✓
3	KR3	Khewra Road	32.6134	73.0239	1×10^{-3}	✓	✓
4	KR4	Khewra Road	32.6134	73.0239	1×10^{-3}	✓	✓
5	KR5	Khewra Road	32.6134	73.0239	1×10^{-5}	✓	✓
6	KR6	Khewra Road	32.6134	73.0239	1×10^{-3}	✓	✓
7	CSKa1	Choa Saidan Shah-Kallar Kahar Road	30.4319	71.9958	1×10^{-3}	✓	✓
8	CSKa2	Choa Saidan Shah-Kallar Kahar Road	30.4319	71.9958	1×10^{-3}	✓	✓
9	CSKa3	Choa Saidan Shah-Kallar Kahar Road	30.4319	71.9958	1×10^{-3}	✓	✓
10	CSKa4	Choa Saidan Shah-Kallar Kahar Road	30.4319	71.9958	1×10^{-3}	✓	✓
11	CSKa5	Choa Saidan Shah-Kallar Kahar Road	30.4319	71.9958	1×10^{-3}	✓	✓
12	CSKa6	Choa Saidan Shah-Kallar Kahar Road	30.4319	71.9958	1×10^{-5}	✓	✓
13	CSKa7	Choa Saidan Shah-Kallar Kahar Road	30.4319	71.9958	1×10^{-5}	✓	✓
14	CSKa8	Choa Saidan Shah-Kallar Kahar Road	30.4319	71.9958	1×10^{-5}	✓	✓
15	CSKa9	Choa Saidan Shah-Kallar Kahar Road	30.4319	71.9958	1×10^{-5}	✓	✓
16	CSKb1	Choa Saidan Shah-Kallar Kahar Road	32.7664	72.7341	1×10^{-3}	✓	✓
17	CSKb2	Choa Saidan Shah-Kallar Kahar Road	32.7664	72.7341	1×10^{-3}	✓	✓
18	CSKb3	Choa Saidan Shah-Kallar Kahar Road	32.7664	72.7341	1×10^{-3}	✓	✓
19	CSKb4	Choa Saidan Shah-Kallar Kahar Road	32.7664	72.7341	1×10^{-5}	✓	✓

20	CSKb5	Choa Saidan Shah-Kallar Kahar Road	32.7664	72.7341	$1×10^{-5}$	✓	✓
21	CSKb6	Choa Saidan Shah-Kallar Kahar Road	32.7664	72.7341	$1×10^{-5}$	✓	✓
22	CSKb7	Choa Saidan Shah-Kallar Kahar Road	32.7664	72.7341	$1×10^{-5}$	✓	✓
23	CSKb8	Choa Saidan Shah-Kallar Kahar Road	32.7664	72.7341	$1×10^{-5}$	✓	✓
24	CSKc1	Choa Saidan Shah-Kallar Kahar Road	32.7655	72.738	$1×10^{-3}$	✓	✓
25	CSKc2	Choa Saidan Shah-Kallar Kahar Road	32.7655	72.738	$1×10^{-3}$	✓	✓
26	CSKc3	Choa Saidan Shah-Kallar Kahar Road	32.7655	72.738	$1×10^{-3}$	✓	✓
27	CSKc4	Choa Saidan Shah-Kallar Kahar Road	32.7655	72.738	$1×10^{-3}$	✓	✓
28	CSKc5	Choa Saidan Shah-Kallar Kahar Road	32.7655	72.738	$1×10^{-3}$	✓	✓
29	CSKc6	Choa Saidan Shah-Kallar Kahar Road	32.7655	72.738	$1×10^{-3}$	✓	✓
**30**	**CSKc7**	**Choa Saidan Shah-Kallar Kahar Road**	**32.7655**	**72.738**	$\mathbf{1×10^{-5}}$	✓	✓
31	CSKc8	Choa Saidan Shah-Kallar Kahar Road	32.7655	72.738	$1×10^{-5}$	✓	✓
32	CSKc9	Choa Saidan Shah-Kallar Kahar Road	32.7655	72.738	$1×10^{-5}$	✓	✓
33	CSKc10	Choa Saidan Shah-Kallar Kahar Road	32.7655	72.738	$1×10^{-5}$	✓	✓
34	CSKd1	Choa Saidan Shah-Kallar Kahar Road	32.7485	72.7574	$1×10^{-3}$	✓	✓
35	CSKd2	Choa Saidan Shah-Kallar Kahar Road	32.7485	72.7574	$1×10^{-3}$	✓	✓
36	CSKd3	Choa Saidan Shah-Kallar Kahar Road	32.7485	72.7574	$1×10^{-3}$	✓	✓
37	CSKd4	Choa Saidan Shah-Kallar Kahar Road	32.7485	72.7574	$1×10^{-3}$	✓	✓
38	CSKd5	Choa Saidan Shah-Kallar Kahar Road	32.7485	72.7574	$1×10^{-5}$	✓	✓
39	CSKd6	Choa Saidan Shah-Kallar Kahar Road	32.7485	72.7574	$1×10^{-5}$	✓	✓
40	CSKd7	Choa Saidan Shah-Kallar Kahar Road	32.7485	72.7574	$1×10^{-5}$	✓	✓
41	CSKd8	Choa Saidan Shah-Kallar Kahar Road	32.7485	72.7574	$1×10^{-5}$	✓	✓
42	CSKd9	Choa Saidan Shah-Kallar Kahar Road	32.7485	72.7574	$1×10^{-5}$	✓	✓

43	CSKd10	Choa Saidan Shah-Kallar Kahar Road	32.7485	72.7574	1×10^{-5}	✓	✓
44	CSKd11	Choa Saidan Shah-Kallar Kahar Road	32.7485	72.7574	1×10^{-5}	✓	✓
45	TKRa1	Talagang-Kallar Kahar Road	32.7863	72.6968	1×10^{-3}	✓	✓
46	TKRa2	Talagang-Kallar Kahar Road	32.7863	72.6968	1×10^{-3}	✓	✓
47	TKRa3	Talagang-Kallar Kahar Road	32.7863	72.6968	1×10^{-3}	✓	✓
48	TKRa4	Talagang-Kallar Kahar Road	32.7863	72.6968	1×10^{-3}	✓	✓
49	TKRa5	Talagang-Kallar Kahar Road	32.7863	72.6968	1×10^{-5}	✓	✓
50	TKRa6	Talagang-Kallar Kahar Road	32.7863	72.6968	1×10^{-5}	✓	✓
51	TKRa7	Talagang-Kallar Kahar Road	32.7863	72.6968	1×10^{-5}	✓	✓
52	TKRa8	Talagang-Kallar Kahar Road	32.7863	72.6968	1×10^{-5}	✓	✓
53	TKRa9	Talagang-Kallar Kahar Road	32.7863	72.6968	1×10^{-5}	✓	✓
54	TKRa10	Talagang-Kallar Kahar Road	32.7863	72.6968	1×10^{-5}	✓	✓
55	TKRa11	Talagang-Kallar Kahar Road	32.7863	72.6968	1×10^{-5}	✓	✓
56	TKRa12	Talagang-Kallar Kahar Road	32.7863	72.6968	1×10^{-5}	✓	✓
57	TKRb1	Talagang-Kallar Kahar Road	32.7885	72.6823	1×10^{-3}	✓	✓
58	TKRb2	Talagang-Kallar Kahar Road	32.7885	72.6823	1×10^{-3}	✓	✓
59	TKRb3	Talagang-Kallar Kahar Road	32.7885	72.6823	1×10^{-3}	✓	✓
60	TKRb4	Talagang-Kallar Kahar Road	32.7885	72.6823	1×10^{-3}	✓	✓
61	TKRb5	Talagang-Kallar Kahar Road	32.7885	72.6823	1×10^{-3}	✓	✓
62	TKRb6	Talagang-Kallar Kahar Road	32.7885	72.6823	1×10^{-3}	✓	✓
63	TKRb7	Talagang-Kallar Kahar Road	32.7885	72.6823	1×10^{-3}	✓	✓
64	TKRb8	Talagang-Kallar Kahar Road	32.7885	72.6823	1×10^{-3}	✓	✓
65	TKRb9	Talagang-Kallar Kahar Road	32.7885	72.6823	1×10^{-5}	✓	✓

66	TKRb10	Talagang-Kallar Kahar Road	32.7885	72.6823	1×10^{-5}	✓	✓
67	TKRb11	Talagang-Kallar Kahar Road	32.7885	72.6823	1×10^{-5}	✓	✓
68	LRa1	Lillah Road	32.5791	72.8075	1×10^{-3}	✓	✓
69	LRa2	Lillah Road	32.5791	72.8075	1×10^{-3}	✓	✓
70	LRa3	Lillah Road	32.5791	72.8075	1×10^{-3}	✓	✓
71	LRa4	Lillah Road	32.5791	72.8075	1×10^{-3}	✓	✓
72	LRa5	Lillah Road	32.5791	72.8075	1×10^{-3}	✓	✓
73	LRa6	Lillah Road	32.5791	72.8075	1×10^{-3}	✓	✓
74	LRa7	Lillah Road	32.5791	72.8075	1×10^{-5}	✓	✓
75	LRa8	Lillah Road	32.5791	72.8075	1×10^{-5}	✓	✓
76	LRa9	Lillah Road	32.5791	72.8075	1×10^{-5}	✓	✓
77	LRa10	Lillah Road	32.5791	72.8075	1×10^{-5}	✓	✓
78	LRa11	Lillah Road	32.5791	72.8075	1×10^{-5}	✓	✓
79	LRb1	Lillah Road	32.5851	72.8931	1×10^{-3}	✓	✓
80	LRb2	Lillah Road	32.5851	72.8931	1×10^{-3}	✓	✓
81	LRb3	Lillah Road	32.5851	72.8931	1×10^{-3}	✓	✓
82	LRb4	Lillah Road	32.5851	72.8931	1×10^{-3}	✓	✓
83	LRb5	Lillah Road	32.5851	72.8931	1×10^{-3}	✓	✓
84	LRb6	Lillah Road	32.5851	72.8931	1×10^{-3}	✓	✓
85	LRb7	Lillah Road	32.5851	72.8931	1×10^{-3}	✓	✓
86	LRb8	Lillah Road	32.5851	72.8931	1×10^{-3}	✓	✓
87	LRb9	Lillah Road	32.5851	72.8931	1×10^{-3}	✓	✓
88	LRb10	Lillah Road	32.5851	72.8931	1×10^{-3}	✓	✓

89	LRb11	Lillah Road	32.5851	72.8931	1×10^{-5}	✓	✓
90	LRb12	Lillah Road	32.5851	72.8931	1×10^{-5}	✓	✓
91	LRb13	Lillah Road	32.5851	72.8931	1×10^{-5}	✓	✓
92	LRb14	Lillah Road	32.5851	72.8931	1×10^{-5}	✓	✓
93	LRb15	Lillah Road	32.5851	72.8931	1×10^{-5}	✓	✓
94	LRb16	Lillah Road	32.5851	72.8931	1×10^{-5}	✓	✓
95	LRc1	Lillah Road	32.5936	73.027	1×10^{-3}	✓	✓
96	LRc2	Lillah Road	32.5936	73.027	1×10^{-3}	✓	✓
97	LRc3	Lillah Road	32.5936	73.027	1×10^{-3}	✓	✓
98	LRc4	Lillah Road	32.5936	73.027	1×10^{-3}	✓	✓
99	LRc5	Lillah Road	32.5936	73.027	1×10^{-3}	✓	✓
100	LRc6	Lillah Road	32.5936	73.027	1×10^{-3}	✓	✓
101	LRc7	Lillah Road	32.5936	73.027	1×10^{-5}	✓	✓
102	LRc8	Lillah Road	32.5936	73.027	1×10^{-5}	✓	✓
103	LRc9	Lillah Road	32.5936	73.027	1×10^{-5}	✓	✓
104	LRc10	Lillah Road	32.5936	73.027	1×10^{-5}	✓	✓
105	LRc11	Lillah Road	32.5936	73.027	1×10^{-5}	✓	✓
106	LRc12	Lillah Road	32.5936	73.027	1×10^{-5}	✓	✓
107	LRc13	Lillah Road	32.5936	73.027	1×10^{-5}	✓	✓
108	LRc14	Lillah Road	32.5936	73.027	1×10^{-5}	✓	✓
109	LRc15	Lillah Road	32.5936	73.027	1×10^{-5}	✓	✓
110	GPB1	Ghubrika, Pindi Bhattiyan	31.8173	73.20805	1×10^{-3}	✓	✓
111	GPB2	Ghubrika, Pindi Bhattiyan	31.8173	73.20805	1×10^{-3}	✓	✓

112	GPB3	Ghubrika, Pindi Bhattiyan	31.8173	73.20805	1×10^{-3}	✓	✓
113	GPB4	Ghubrika, Pindi Bhattiyan	31.8173	73.20805	1×10^{-3}	✓	✓
114	GPB5	Ghubrika, Pindi Bhattiyan	31.8173	73.20805	1×10^{-3}	✓	✓
115	GPB6	Ghubrika, Pindi Bhattiyan	31.8173	73.20805	1×10^{-3}	✓	✓
116	GPB7	Ghubrika, Pindi Bhattiyan	31.8173	73.20805	1×10^{-3}	✓	✓
117	GPB8	Ghubrika, Pindi Bhattiyan	31.8173	73.20805	1×10^{-3}	✓	✓
118	GPB9	Ghubrika, Pindi Bhattiyan	31.8173	73.20805	1×10^{-3}	✓	✓
119	GPB10	Ghubrika, Pindi Bhattiyan	31.8173	73.20805	1×10^{-3}	✓	✓
120	GPB11	Ghubrika, Pindi Bhattiyan	31.8173	73.20805	1×10^{-3}	✓	✓
121	GPB12	Ghubrika, Pindi Bhattiyan	31.8173	73.20805	1×10^{-3}	✓	✓
122	GPB13	Ghubrika, Pindi Bhattiyan	31.8173	73.20805	1×10^{-5}	✓	✓
123	GPB14	Ghubrika, Pindi Bhattiyan	31.8173	73.20805	1×10^{-5}	✓	✓
124	GPB15	Ghubrika, Pindi Bhattiyan	31.8173	73.20805	1×10^{-5}	✓	✓
125	GPB16	Ghubrika, Pindi Bhattiyan	31.8173	73.20805	1×10^{-5}	✓	✓
126	GPB17	Ghubrika, Pindi Bhattiyan	31.8173	73.20805	1×10^{-5}	✓	✓
127	GPB18	Ghubrika, Pindi Bhattiyan	31.8173	73.20805	1×10^{-5}	✓	✓
128	GPB19	Ghubrika, Pindi Bhattiyan	31.8173	73.20805	1×10^{-5}	✓	✓
129	GPB20	Ghubrika, Pindi Bhattiyan	31.8173	73.20805	1×10^{-5}	✓	✓
130	GPB21	Ghubrika, Pindi Bhattiyan	31.8173	73.20805	1×10^{-5}	✓	✓
131	BWC1	Burjian Wala, Chiniot	31.8055	73.1547	1×10^{-3}	✓	✓
132	BWC2	Burjian Wala, Chiniot	31.8055	73.1547	1×10^{-3}	✓	✓
133	BWC3	Burjian Wala, Chiniot	31.8055	73.1547	1×10^{-3}	✓	✓
134	BWC4	Burjian Wala, Chiniot	31.8055	73.1547	1×10^{-3}	✓	✓

135	BWC5	Burjian Wala, Chiniot	31.8055	73.1547	1×10^{-3}	✓	✓
136	BWC6	Burjian Wala, Chiniot	31.8055	73.1547	1×10^{-3}	✓	✓
137	BWC7	Burjian Wala, Chiniot	31.8055	73.1547	1×10^{-3}	✓	✓
138	BWC8	Burjian Wala, Chiniot	31.8055	73.1547	1×10^{-3}	✓	✓
139	BWC9	Burjian Wala, Chiniot	31.8055	73.1547	1×10^{-3}	✓	✓
140	BWC10	Burjian Wala, Chiniot	31.8055	73.1547	1×10^{-3}	✓	✓
141	BWC11	Burjian Wala, Chiniot	31.8055	73.1547	1×10^{-3}	✓	✓
142	BWC12	Burjian Wala, Chiniot	31.8055	73.1547	1×10^{-3}	✓	✓
143	BWC13	Burjian Wala, Chiniot	31.8055	73.1547	1×10^{-3}	✓	✓
144	BWC14	Burjian Wala, Chiniot	31.8055	73.1547	1×10^{-3}	✓	✓
145	BWC15	Burjian Wala, Chiniot	31.8055	73.1547	1×10^{-5}	✓	✓
146	BWC16	Burjian Wala, Chiniot	31.8055	73.1547	1×10^{-5}	✓	✓
147	BWC17	Burjian Wala, Chiniot	31.8055	73.1547	1×10^{-5}	✓	✓
148	BWC18	Burjian Wala, Chiniot	31.8055	73.1547	1×10^{-5}	✓	✓
149	BWC19	Burjian Wala, Chiniot	31.8055	73.1547	1×10^{-5}	✓	✓
150	BWC20	Burjian Wala, Chiniot	31.8055	73.1547	1×10^{-5}	✓	✓
151	BWC21	Burjian Wala, Chiniot	31.8055	73.1547	1×10^{-5}	✓	✓
152	BWC22	Burjian Wala, Chiniot	31.8055	73.1547	1×10^{-5}	✓	✓
153	BWC23	Burjian Wala, Chiniot	31.8055	73.1547	1×10^{-5}	✓	✓
154	TTS1	Toba Tek Singh	30.9558	72.4766	1×10^{-3}	✓	✓
155	TTS2	Toba Tek Singh	30.9558	72.4766	1×10^{-3}	✓	✓
156	TTS3	Toba Tek Singh	30.9558	72.4766	1×10^{-3}	✓	✓
157	TTS4	Toba Tek Singh	30.9558	72.4766	1×10^{-3}	✓	✓

158	TTS5	Toba Tek Singh	30.9558	72.4766	1×10^{-3}	✓	✓
159	TTS6	Toba Tek Singh	30.9558	72.4766	1×10^{-3}	✓	✓
160	TTS7	Toba Tek Singh	30.9558	72.4766	1×10^{-3}	✓	✓
161	TTS8	Toba Tek Singh	30.9558	72.4766	1×10^{-3}	✓	✓
162	TTS9	Toba Tek Singh	30.9558	72.4766	1×10^{-3}	✓	✓
163	TTS10	Toba Tek Singh	30.9558	72.4766	1×10^{-3}	✓	✓
164	TTS11	Toba Tek Singh	30.9558	72.4766	1×10^{-3}	✓	✓
165	TTS12	Toba Tek Singh	30.9558	72.4766	1×10^{-3}	✓	✓
166	TTS13	Toba Tek Singh	30.9558	72.4766	1×10^{-3}	✓	✓
167	TTS14	Toba Tek Singh	30.9558	72.4766	1×10^{-3}	✓	✓
168	TTS15	Toba Tek Singh	30.9558	72.4766	1×10^{-3}	✓	✓
169	TTS16	Toba Tek Singh	30.9558	72.4766	1×10^{-3}	✓	✓
170	TTS17	Toba Tek Singh	30.9558	72.4766	1×10^{-5}	✓	✓
171	TTS18	Toba Tek Singh	30.9558	72.4766	1×10^{-5}	✓	✓
172	TTS19	Toba Tek Singh	30.9558	72.4766	1×10^{-5}	✓	✓
173	TTS20	Toba Tek Singh	30.9558	72.4766	1×10^{-5}	✓	✓
174	TTS21	Toba Tek Singh	30.9558	72.4766	1×10^{-5}	✓	✓
175	TTS22	Toba Tek Singh	30.9558	72.4766	1×10^{-5}	✓	✓
176	Dw1	Dolu-Wala	31.8026	73.1768	1×10^{-3}	✓	✓
177	Dw2	Dolu-Wala	31.8026	73.1768	1×10^{-3}	✓	✓
178	Dw3	Dolu-Wala	31.8026	73.1768	1×10^{-3}	✓	✓
179	Dw4	Dolu-Wala	31.8026	73.1768	1×10^{-3}	✓	✓
180	Dw5	Dolu-Wala	31.8026	73.1768	1×10^{-3}	✓	✓

181	Dw6	Dolu-Wala	31.8026	73.1768	1×10^{-3}	✓	✓
182	Dw7	Dolu-Wala	31.8026	73.1768	1×10^{-3}	✓	✓
183	Dw8	Dolu-Wala	31.8026	73.1768	1×10^{-3}	✓	✓
184	Dw9	Dolu-Wala	31.8026	73.1768	1×10^{-3}	✓	✓
185	Dw10	Dolu-Wala	31.8026	73.1768	1×10^{-5}	✓	✓
186	Dw11	Dolu-Wala	31.8026	73.1768	1×10^{-5}	✓	✓
187	Dw12	Dolu-Wala	31.8026	73.1768	1×10^{-5}	✓	✓
188	Dw13	Dolu-Wala	31.8026	73.1768	1×10^{-5}	✓	✓
189	Dw14	Dolu-Wala	31.8026	73.1768	1×10^{-5}	✓	✓
190	Dw15	Dolu-Wala	31.8026	73.1768	1×10^{-5}	✓	✓
191	Dw16	Dolu-Wala	31.8026	73.1768	1×10^{-5}	✓	✓
192	Dw17	Dolu-Wala	31.8026	73.1768	1×10^{-5}	✓	✓
193	Dw18	Dolu-Wala	31.8026	73.1768	1×10^{-5}	✓	✓
194	PA1	Pakka Anna	31.2455	72.7981	1×10^{-3}	✓	✓
195	PA2	Pakka Anna	31.2455	72.7981	1×10^{-3}	✓	✓
196	PA3	Pakka Anna	31.2455	72.7981	1×10^{-3}	✓	✓
197	PA4	Pakka Anna	31.2455	72.7981	1×10^{-3}	✓	✓
198	PA5	Pakka Anna	31.2455	72.7981	1×10^{-3}	✓	✓
199	PA6	Pakka Anna	31.2455	72.7981	1×10^{-3}	✓	✓
200	PA7	Pakka Anna	31.2455	72.7981	1×10^{-3}	✓	✓
201	PA8	Pakka Anna	31.2455	72.7981	1×10^{-3}	✓	✓
202	PA9	Pakka Anna	31.2455	72.7981	1×10^{-3}	✓	✓
203	PA10	Pakka Anna	31.2455	72.7981	1×10^{-3}	✓	✓

204	PA11	Pakka Anna	31.2455	72.7981	1×10^{-3}	✓	✓
205	PA12	Pakka Anna	31.2455	72.7981	1×10^{-3}	✓	✓
206	PA13	Pakka Anna	31.2455	72.7981	1×10^{-3}	✓	✓
207	PA14	Pakka Anna	31.2455	72.7981	1×10^{-3}	✓	✓
208	PA15	Pakka Anna	31.2455	72.7981	1×10^{-5}	✓	✓
209	PA16	Pakka Anna	31.2455	72.7981	1×10^{-5}	✓	✓
210	PA17	Pakka Anna	31.2455	72.7981	1×10^{-5}	✓	✓
211	PA18	Pakka Anna	31.2455	72.7981	1×10^{-5}	✓	✓
212	PA19	Pakka Anna	31.2455	72.7981	1×10^{-5}	✓	✓
213	PA20	Pakka Anna	31.2455	72.7981	1×10^{-5}	✓	✓
214	PA21	Pakka Anna	31.2455	72.7981	1×10^{-5}	✓	✓
215	PA22	Pakka Anna	31.2455	72.7981	1×10^{-5}	✓	✓
216	PA23	Pakka Anna	31.2455	72.7981	1×10^{-5}	✓	✓
217	PA24	Pakka Anna	31.2455	72.7981	1×10^{-5}	✓	✓
218	PA25	Pakka Anna	31.2455	72.7981	1×10^{-5}	✓	✓
219	PA26	Pakka Anna	31.2455	72.7981	1×10^{-5}	✓	✓
220	PA27	Pakka Anna	31.2455	72.7981	1×10^{-5}	✓	✓
221	PA28	Pakka Anna	31.2455	72.7981	1×10^{-5}	✓	✓
222	PA29	Pakka Anna	31.2455	72.7981	1×10^{-5}	✓	✓
223	PA30	Pakka Anna	31.2455	72.7981	1×10^{-5}	✓	✓
224	PA31	Pakka Anna	31.2455	72.7981	1×10^{-5}	✓	✓
225	PA32	Pakka Anna	31.2455	72.7981	1×10^{-5}	✓	✓
226	PA33	Pakka Anna	31.2455	72.7981	1×10^{-5}	✓	✓

227	PA34	Pakka Anna	31.2455	72.7981	1×10^{-5}	✓	✓
228	PA35	Pakka Anna	31.2455	72.7981	1×10^{-5}	✓	✓
229	PA36	Pakka Anna	31.2455	72.7981	1×10^{-5}	✓	✓
230	PA37	Pakka Anna	31.2455	72.7981	1×10^{-5}	✓	✓
231	DLG1	Dad Leghari, Ghotki	27.9474	69.6663	1×10^{-3}	✓	✓
232	DLG2	Dad Leghari, Ghotki	27.9474	69.6663	1×10^{-3}	✓	✓
233	DLG3	Dad Leghari, Ghotki	27.9474	69.6663	1×10^{-3}	✓	✓
234	DLG4	Dad Leghari, Ghotki	27.9474	69.6663	1×10^{-3}	✓	✓
235	DLG5	Dad Leghari, Ghotki	27.9474	69.6663	1×10^{-3}	✓	✓
236	DLG6	Dad Leghari, Ghotki	27.9474	69.6663	1×10^{-3}	✓	✓
237	DLG7	Dad Leghari, Ghotki	27.9474	69.6663	1×10^{-3}	✓	✓
238	DLG8	Dad Leghari, Ghotki	27.9474	69.6663	1×10^{-3}	✓	✓
239	DLG9	Dad Leghari, Ghotki	27.9474	69.6663	1×10^{-3}	✓	✓
240	DLG10	Dad Leghari, Ghotki	27.9474	69.6663	1×10^{-3}	✓	✓
241	DLG11	Dad Leghari, Ghotki	27.9474	69.6663	1×10^{-3}	✓	✓
242	DLG12	Dad Leghari, Ghotki	27.9474	69.6663	1×10^{-3}	✓	✓
243	DLG13	Dad Leghari, Ghotki	27.9474	69.6663	1×10^{-3}	✓	✓
244	DLG14	Dad Leghari, Ghotki	27.9474	69.6663	1×10^{-3}	✓	✓
245	DLG15	Dad Leghari, Ghotki	27.9474	69.6663	1×10^{-3}	✓	✓
246	DLG16	Dad Leghari, Ghotki	27.9474	69.6663	1×10^{-3}	✓	✓
247	DLG17	Dad Leghari, Ghotki	27.9474	69.6663	1×10^{-5}	✓	✓
248	DLG18	Dad Leghari, Ghotki	27.9474	69.6663	1×10^{-5}	✓	✓
249	DLG19	Dad Leghari, Ghotki	27.9474	69.6663	1×10^{-5}	✓	✓

250	DLG20	Dad Leghari, Ghotki	27.9474	69.6663	1×10^{-5}	✓	✓
251	DLG21	Dad Leghari, Ghotki	27.9474	69.6663	1×10^{-5}	✓	✓
252	DLG22	Dad Leghari, Ghotki	27.9474	69.6663	1×10^{-5}	✓	✓
253	DLG23	Dad Leghari, Ghotki	27.9474	69.6663	1×10^{-5}	✓	✓
254	DLG24	Dad Leghari, Ghotki	27.9474	69.6663	1×10^{-5}	✓	✓
255	DLG25	Dad Leghari, Ghotki	27.9474	69.6663	1×10^{-5}	✓	✓
256	DLG26	Dad Leghari, Ghotki	27.9474	69.6663	1×10^{-5}	✓	✓
257	DLG27	Dad Leghari, Ghotki	27.9474	69.6663	1×10^{-5}	✓	✓
258	DLG28	Dad Leghari, Ghotki	27.9474	69.6663	1×10^{-5}	✓	✓
259	DLG29	Dad Leghari, Ghotki	27.9474	69.6663	1×10^{-5}	✓	✓
260	DLG30	Dad Leghari, Ghotki	27.9474	69.6663	1×10^{-5}	✓	✓
261	DLG31	Dad Leghari, Ghotki	27.9474	69.6663	1×10^{-5}	✓	✓
262	DLG32	Dad Leghari, Ghotki	27.9474	69.6663	1×10^{-5}	✓	✓
263	DLG33	Dad Leghari, Ghotki	27.9474	69.6663	1×10^{-5}	✓	✓
264	DLG34	Dad Leghari, Ghotki	27.9474	69.6663	1×10^{-5}	✓	✓
265	BBD1	Bago Bhutto, Daharki	28.0403	69.6561	1×10^{-3}	✓	✓
266	BBD2	Bago Bhutto, Daharki	28.0403	69.6561	1×10^{-3}	✓	✓
267	BBD3	Bago Bhutto, Daharki	28.0403	69.6561	1×10^{-3}	✓	✓
268	BBD4	Bago Bhutto, Daharki	28.0403	69.6561	1×10^{-3}	✓	✓
269	BBD5	Bago Bhutto, Daharki	28.0403	69.6561	1×10^{-3}	✓	✓
270	BBD6	Bago Bhutto, Daharki	28.0403	69.6561	1×10^{-3}	✓	✓
271	BBD7	Bago Bhutto, Daharki	28.0403	69.6561	1×10^{-3}	✓	✓
272	BBD8	Bago Bhutto, Daharki	28.0403	69.6561	1×10^{-3}	✓	✓

273	BBD9	Bago Bhutto, Daharki	28.0403	69.6561	1×10^{-3}	✓	✓
274	BBD10	Bago Bhutto, Daharki	28.0403	69.6561	1×10^{-3}	✓	✓
275	BBD11	Bago Bhutto, Daharki	28.0403	69.6561	1×10^{-3}	✓	✓
276	BBD12	Bago Bhutto, Daharki	28.0403	69.6561	1×10^{-3}	✓	✓
277	BBD13	Bago Bhutto, Daharki	28.0403	69.6561	1×10^{-3}	✓	✓
278	BBD14	Bago Bhutto, Daharki	28.0403	69.6561	1×10^{-3}	✓	✓
279	BBD15	Bago Bhutto, Daharki	28.0403	69.6561	1×10^{-3}	✓	✓
280	BBD16	Bago Bhutto, Daharki	28.0403	69.6561	1×10^{-3}	✓	✓
281	BBD17	Bago Bhutto, Daharki	28.0403	69.6561	1×10^{-3}	✓	✓
282	BBD18	Bago Bhutto, Daharki	28.0403	69.6561	1×10^{-3}	✓	✓
283	BBD19	Bago Bhutto, Daharki	28.0403	69.6561	1×10^{-3}	✓	✓
284	BBD20	Bago Bhutto, Daharki	28.0403	69.6561	1×10^{-3}	✓	✓
285	BBD21	Bago Bhutto, Daharki	28.0403	69.6561	1×10^{-5}	✓	✓
286	BBD22	Bago Bhutto, Daharki	28.0403	69.6561	1×10^{-5}	✓	✓
287	BBD23	Bago Bhutto, Daharki	28.0403	69.6561	1×10^{-5}	✓	✓
288	BBD24	Bago Bhutto, Daharki	28.0403	69.6561	1×10^{-5}	✓	✓
289	BBD25	Bago Bhutto, Daharki	28.0403	69.6561	1×10^{-5}	✓	✓
290	BBD26	Bago Bhutto, Daharki	28.0403	69.6561	1×10^{-5}	✓	✓
291	BBD27	Bago Bhutto, Daharki	28.0403	69.6561	1×10^{-5}	✓	✓
292	BBD28	Bago Bhutto, Daharki	28.0403	69.6561	1×10^{-5}	✓	✓
293	BBD29	Bago Bhutto, Daharki	28.0403	69.6561	1×10^{-5}	✓	✓
294	BBD30	Bago Bhutto, Daharki	28.0403	69.6561	1×10^{-5}	✓	✓
295	BBD31	Bago Bhutto, Daharki	28.0403	69.6561	1×10^{-5}	✓	✓

296	BBD32	Bago Bhutto, Daharki	28.0403	69.6561	1×10^{-5}	✓	✓
297	BBD33	Bago Bhutto, Daharki	28.0403	69.6561	1×10^{-5}	✓	✓

Supplementary Table 2: Determination of minimum inhibitory concentration (MIC) for NaCl of bacterial isolates on salt supplemented media

Sr. No.	Isolates Initial Code	Final Code	LB media (0.5% NaCl)	1% NaCl	5% NaCl	7% NaCl	10% NaCl
1	CSKa1	UM1	✓	✓	✓	✓	×
2	CSKa2	UM2	✓	✓	✓	✓	✓
3	CSKa3		✓	✓	×	×	×
4	CSKa4	UM3	✓	✓	✓	✓	×
5	CSKa5		✓	✓	×	×	×
6	CSKa6	UM4	✓	✓	✓	✓	✓
7	CSKa7		✓	✓	✓	×	×
8	CSKa8	UM5	✓	✓	✓	✓	✓
9	CSKa9	UM5	✓	✓	✓	✓	✓
10	KR1	UM6	✓	✓	✓	✓	✓
11	KR2		✓	✓	×	×	×
12	KR3		✓	✓	✓	×	×
13	KR4	UM7	✓	✓	✓	✓	×
14	KR5		✓	✓	×	×	×
15	KR6	UM8	✓	✓	✓	✓	✓
16	CSKb1	UM9	✓	✓	✓	✓	✓
17	CSKb2		✓	✓	×	×	×
18	CSKb3	UM10	✓	✓	✓	✓	×
19	CSKb4		✓	✓	×	×	×
20	CSKb5		✓	✓	✓	×	×

21	CSKb6	UM11	✓	✓	✓	✓	✓
22	CSKb7		✓	✓	×	×	×
23	CSKb8		✓	✓	×	×	×
24	CSKc1	UM12	✓	✓	✓	✓	×
25	CSKc2	UM13	✓	✓	✓	✓	✓
26	CSKc3	UM14	✓	✓	✓	✓	✓
27	CSKc4	UM15	✓	✓	✓	✓	✓
28	CSKc5		✓	✓	✓	×	×
29	CSKc6		✓	✓	✓	×	×
30	CSKc7	UM16	✓	✓	✓	✓	✓
31	CSKc8		✓	✓	×	×	×
32	CSKc9	UM17	✓	✓	✓	✓	✓
33	CSKc10		✓	✓	✓	×	×
34	CSKd1		✓	✓	×	×	×
35	CSKd2	UM18	✓	✓	✓	✓	✓
36	CSKd3	UM19	✓	✓	✓	✓	×
37	CSKd4	UM20	✓	✓	✓	✓	✓
38	CSKd5		✓	✓	×	×	×
39	CSKd6		✓	✓	×	×	×
40	CSKd7	UM21	✓	✓	✓	✓	×
41	CSKd8	UM22	✓	✓	✓	✓	✓
42	CSKd9		✓	✓	✓	×	×
43	CSKd10	UM23	✓	✓	✓	✓	✓
44	CSKd11		✓	✓	×	×	×
45	TKRa1	UM24	✓	✓	✓	✓	✓
46	TKRa2	UM25	✓	✓	✓	✓	✓
47	TKRa3		✓	✓	×	×	×

48	TKRa4		✓	✓	×	×	×
49	TKRa5	UM26	✓	✓	✓	✓	×
50	TKRa6	UM27	✓	✓	✓	✓	✓
51	TKRa7	UM28	✓	✓	✓	✓	✓
52	TKRa8	UM29	✓	✓	✓	✓	×
53	TKRa9		✓	✓	✓	×	×
54	TKRa10	UM30	✓	✓	✓	✓	✓
55	TKRa11		✓	✓	×	×	×
56	TKRa12	UM31	✓	✓	✓	✓	✓
57	TKRb1		✓	✓	×	×	×
58	TKRb2	UM32	✓	✓	✓	✓	✓
59	TKRb3	UM33	✓	✓	✓	✓	×
60	TKRb4		✓	✓	✓	×	×
61	TKRb5		✓	✓	×	×	×
62	TKRb6	UM34	✓	✓	✓	✓	×
63	TKRb7	UM35	✓	✓	✓	✓	✓
64	TKRb8	UM36	✓	✓	✓	✓	✓
65	TKRb9		✓	✓	×	×	×
66	TKRb10	UM37	✓	✓	✓	✓	✓
67	TKRb11		✓	✓	×	×	×
68	LRa1	UM38	✓	✓	✓	✓	✓
69	LRa2	UM39	✓	✓	✓	✓	×
70	LRa3		✓	✓	×	×	×
71	LRa4	UM40	✓	✓	✓	✓	✓
72	LRa5		✓	✓	✓	×	×
73	LRa6		✓	✓	×	×	×
74	LRa7	UM41	✓	✓	✓	✓	✓

75	LRa8	UM42	✓	✓	✓	✓	✓
76	LRa9	UM43	✓	✓	✓	✓	×
77	LRa10		✓	✓	×	×	×
78	LRa11	UM44	✓	✓	✓	✓	×
79	LRb1	UM45	✓	✓	✓	✓	✓
80	LRb2		✓	✓	✓	×	×
81	LRb3	UM46	✓	✓	✓	✓	✓
82	LRb4		✓	✓	×	×	×
83	LRb5		✓	✓	×	×	×
84	LRb6	UM47	✓	✓	✓	✓	✓
85	LRb7	UM48	✓	✓	✓	✓	✓
86	LRb8		✓	✓	×	×	×
87	LRb9	UM49	✓	✓	✓	✓	×
88	LRb10	UM50	✓	✓	✓	✓	✓
89	LRb11	UM51	✓	✓	✓	✓	✓
90	LRb12		✓	✓	×	×	×
91	LRb13	UM52	✓	✓	✓	✓	✓
92	LRb14		✓	✓	×	×	×
93	LRb15	UM53	✓	✓	✓	✓	✓
94	LRb16	UM54	✓	✓	✓	✓	✓
95	LRc1		✓	✓	×	×	×
96	LRc2		✓	✓	×	×	×
97	LRc3	UM55	✓	✓	✓	✓	×
98	LRc4		✓	✓	×	×	×
99	LRc5	UM56	✓	✓	✓	✓	✓
100	LRc6	UM57	✓	✓	✓	✓	✓
101	LRc7	UM58	✓	✓	✓	✓	✓

102	LRc8		✓	✓	×	×	×
103	LRc9		✓	✓	✓	×	×
104	LRc10		✓	✓	✓	×	×
105	LRc11	UM59	✓	✓	✓	✓	×
106	LRc12		✓	✓	×	×	×
107	LRc13		✓	✓	×	×	×
108	LRc14	UM60	✓	✓	✓	✓	×
109	LRc15	UM61	✓	✓	✓	✓	✓
110	GPB1		✓	✓	×	×	×
111	GPB2	UM62	✓	✓	✓	✓	✓
112	GPB3	UM63	✓	✓	✓	✓	×
113	GPB4		✓	✓	×	×	×
114	GPB5	UM64	✓	✓	✓	✓	✓
115	GPB6		✓	✓	×	×	×
116	GPB7		✓	✓	✓	×	×
117	GPB8	UM65	✓	✓	✓	✓	✓
118	GPB9	UM66	✓	✓	✓	✓	✓
119	GPB10	UM67	✓	✓	✓	✓	×
120	GPB11		✓	✓	×	×	×
121	GPB12		✓	✓	✓	×	×
122	GPB13	UM68	✓	✓	✓	✓	×
123	GPB14		✓	✓	×	×	×
124	GPB15	UM69	✓	✓	✓	✓	✓
125	GPB16	UM70	✓	✓	✓	✓	✓
126	GPB17		✓	✓	×	×	×
127	GPB18		✓	✓	✓	×	×
128	GPB19	UM71	✓	✓	✓	✓	×

129	GPB20	UM72	✓	✓	✓	✓	✓
130	GPB21	UM73	✓	✓	✓	✓	✓
131	BWC1	UM74	✓	✓	✓	✓	×
132	BWC2		✓	✓	×	×	×
133	BWC3		✓	✓	×	×	×
134	BWC4	UM75	✓	✓	✓	✓	✓
135	BWC5	UM76	✓	✓	✓	✓	✓
136	BWC6		✓	✓	×	×	×
137	BWC7	UM77	✓	✓	✓	✓	×
138	BWC8		✓	✓	×	×	×
139	BWC9		✓	✓	✓	×	×
140	BWC10		✓	✓	✓	×	×
141	BWC11	UM78	✓	✓	✓	✓	×
142	BWC12		✓	✓	×	×	×
143	BWC13	UM79	✓	✓	✓	✓	✓
144	BWC14		✓	✓	×	×	×
145	BWC15	UM80	✓	✓	✓	✓	✓
146	BWC16	UM81	✓	✓	✓	✓	✓
147	BWC17		✓	✓	×	×	×
148	BWC18		✓	✓	✓	×	×
149	BWC19	UM82	✓	✓	✓	✓	×
150	BWC20		✓	✓	×	×	×
151	BWC21		✓	✓	×	×	×
152	BWC22	UM83	✓	✓	✓	✓	✓
153	BWC23	UM84	✓	✓	✓	✓	×
154	TTS1	UM85	✓	✓	✓	✓	✓
155	TTS2		✓	✓	✓	×	×

156	TTS3		✓	✓	✓	×	×
157	TTS4	UM86	✓	✓	✓	✓	✓
158	TTS5	UM87	✓	✓	✓	✓	✓
159	TTS6		✓	✓	×	×	×
160	TTS7		✓	✓	×	×	×
161	TTS8		✓	✓	×	×	×
162	TTS9	UM88	✓	✓	✓	✓	✓
163	TTS10		✓	✓	×	×	×
164	TTS11		✓	✓	×	×	×
165	TTS12	UM89	✓	✓	✓	✓	×
166	TTS13	UM90	✓	✓	✓	✓	✓
167	TTS14	UM91	✓	✓	✓	✓	✓
168	TTS15	UM92	✓	✓	✓	✓	×
169	TTS16		✓	✓	✓	×	×
170	TTS17		✓	✓	✓	×	×
171	TTS18	UM93	✓	✓	✓	✓	×
172	TTS19		✓	✓	×	×	×
173	TTS20	UM94	✓	✓	✓	✓	✓
174	TTS21		✓	✓	×	×	×
175	TTS22		✓	✓	×	×	×
176	Dw1	UM95	✓	✓	✓	✓	✓
177	Dw2	UM96	✓	✓	✓	✓	✓
178	Dw3	UM97	✓	✓	✓	✓	×
179	Dw4		✓	✓	×	×	×
180	Dw5	UM98	✓	✓	✓	✓	×
181	Dw6		✓	✓	×	×	×
182	Dw7	UM99	✓	✓	✓	✓	✓

183	Dw8	UM100	✓	✓	✓	✓	×
184	Dw9	UM101	✓	✓	✓	✓	×
185	Dw10	UM102	✓	✓	✓	✓	✓
186	Dw11		✓	✓	×	×	×
187	Dw12		✓	✓	✓	×	×
188	Dw13		✓	✓	×	×	×
189	Dw14	UM103	✓	✓	✓	✓	×
190	Dw15	UM104	✓	✓	✓	✓	✓
191	Dw16		✓	✓	✓	×	×
192	Dw17		✓	✓	✓	×	×
193	Dw18	UM105	✓	✓	✓	✓	✓
194	PA1		✓	✓	×	×	×
195	PA2		✓	✓	×	×	×
196	PA3	UM106	✓	✓	✓	✓	✓
197	PA4	UM107	✓	✓	✓	✓	×
198	PA5	UM108	✓	✓	✓	✓	✓
199	PA6		✓	✓	×	×	×
200	PA7	UM109	✓	✓	✓	✓	×
201	PA8	UM110	✓	✓	✓	✓	✓
202	PA9		✓	✓	×	×	×
203	PA10	UM111	✓	✓	✓	✓	✓
204	PA11	UM112	✓	✓	✓	✓	✓
205	PA12	UM113	✓	✓	✓	✓	×
206	PA13		✓	✓	×	×	×
207	PA14		✓	✓	×	×	×
208	PA15	UM114	✓	✓	✓	✓	×
209	PA16		✓	✓	×	×	×

210	PA17	UM115	✓	✓	✓	✓	✓
211	PA18		✓	✓	×	×	×
212	PA19		✓	✓	×	×	×
213	PA20		✓	✓	×	×	×
214	PA21	UM116	✓	✓	✓	✓	×
215	PA22	UM117	✓	✓	✓	✓	×
216	PA23	UM118	✓	✓	✓	✓	✓
217	PA24	UM119	✓	✓	✓	✓	✓
218	PA25		✓	✓	×	×	×
219	PA26		✓	✓	×	×	×
220	PA27	UM120	✓	✓	✓	✓	✓
221	PA28	UM121	✓	✓	✓	✓	×
222	PA29		✓	✓	✓	×	×
223	PA30		✓	✓	×	×	×
224	PA31	UM122	✓	✓	✓	✓	×
225	PA32	UM123	✓	✓	✓	✓	✓
226	PA33	UM124	✓	✓	✓	✓	×
227	PA34		✓	✓	×	×	×
228	PA35		✓	✓	✓	×	×
229	PA36	UM125	✓	✓	✓	✓	✓
230	PA37	UM126	✓	✓	✓	✓	✓
231	DLG1	UM127	✓	✓	✓	✓	×
232	DLG2		✓	✓	×	×	×
233	DLG3	UM128	✓	✓	✓	✓	×
234	DLG4		✓	✓	×	×	×
235	DLG5	UM129	✓	✓	✓	✓	✓
236	DLG6	UM130	✓	✓	✓	✓	✓

237	DLG7		✓	✓	✓	×	×
238	DLG8	UM131	✓	✓	✓	✓	×
239	DLG9		✓	✓	×	×	×
240	DLG10	UM132	✓	✓	✓	✓	✓
241	DLG11		✓	✓	×	×	×
242	DLG12		✓	✓	✓	×	×
243	DLG13		✓	✓	✓	✓	×
244	DLG14	UM133	✓	✓	✓	✓	✓
245	DLG15		✓	✓	✓	✓	×
246	DLG16		✓	✓	×	×	×
247	DLG17	UM134	✓	✓	✓	✓	✓
248	DLG18		✓	✓	×	×	×
249	DLG19	UM135	✓	✓	✓	✓	✓
250	DLG20	UM136	✓	✓	✓	✓	✓
251	DLG21		✓	✓	×	×	×
252	DLG22		✓	✓	×	×	×
253	DLG23	UM137	✓	✓	✓	✓	✓
254	DLG24		✓	✓	✓	×	×
255	DLG25	UM138	✓	✓	✓	✓	✓
256	DLG26		✓	✓	×	×	×
257	DLG27	UM139	✓	✓	✓	✓	✓
258	DLG28		✓	✓	✓	✓	×
259	DLG29	UM140	✓	✓	✓	✓	✓
260	DLG30		✓	✓	✓	✓	×
261	DLG31		✓	✓	✓	×	×
262	DLG32		✓	✓	✓	✓	×
263	DLG33		✓	✓	×	×	×

264	DLG34	UM141	✓	✓	✓	✓	✓
265	BBD1		✓	✓	×	×	×
266	BBD2	UM142	✓	✓	✓	✓	×
267	BBD3	UM143	✓	✓	✓	✓	✓
268	BBD4	UM144	✓	✓	✓	✓	✓
269	BBD5		✓	✓	✓	×	×
270	BBD6		✓	✓	×	×	×
271	BBD7	UM145	✓	✓	✓	✓	×
272	BBD8	UM146	✓	✓	✓	✓	×
273	BBD9		✓	✓	×	×	×
274	BBD10		✓	✓	✓	×	×
275	BBD11	UM147	✓	✓	✓	✓	×
276	BBD12	UM148	✓	✓	✓	✓	✓
277	BBD13		✓	✓	×	×	×
278	BBD14		✓	✓	×	×	×
279	BBD15	UM149	✓	✓	✓	✓	×
280	BBD16	UM150	✓	✓	✓	✓	✓
281	BBD17		✓	✓	×	×	×
282	BBD18	UM151	✓	✓	✓	✓	✓
283	BBD19	UM152	✓	✓	✓	✓	✓
284	BBD20	UM152	✓	✓	✓	✓	×
285	BBD21		✓	✓	✓	×	×
286	BBD22		✓	✓	×	×	×
287	BBD23	UM153	✓	✓	✓	✓	✓
288	BBD24	UM154	✓	✓	✓	✓	×
289	BBD25		✓	✓	×	×	×
290	BBD26		✓	✓	×	×	×

291	BBD27	UM155	✓	✓	✓	✓	✓
292	BBD28	UM156	✓	✓	✓	✓	✓
293	BBD29	UM157	✓	✓	✓	✓	×
294	BBD30		✓	✓	×	×	×
295	BBD31	UM158	✓	✓	✓	✓	×
296	BBD32		✓	✓	×	×	×
297	BBD33	UM159	✓	✓	✓	✓	✓

Supplementary Table 3: Detection for general plant growth promoting (PGP) characteristics and other selected biochemical activities associated directly or indirectly with PGP and bacterial adaptation under saline environment.

Sr. No.	Isolate Code	N_2-fixa-tion	P-solubili-zation	IAA produc-tion	Sidero-phore produ-ction	Zn-solubiliza-tion	EPS produc-tion	HCN produc-tion	Catalase produc-tion	Peroxi-dase produc-tion	Cellu-lose de-gradation
		NFM media	Pikovas-kaya's media	L-trypto-phan	Chrome azrole S media	Alexandrove's media	Culture super-natant + Ethanol				Starch Hydro-lysis
1	UM1	-	-	-	+	+	+	-	+	+	-
2	UM2	-	-	-	-	-	+	-	+	+	-
3	UM3	+	-	-	-	-	+	-	+	+	-
4	UM4	+	-	-	-	-	+	-	+	+	-
5	UM5	-	-	-	+	-	-	-	+	+	+
6	UM6	-	+	-	-	-	-	-	+	+	+
7	UM7	-	-	-	-	-	+	-	+	-	-
8	UM8	-	-	-	-	+	+	-	+	-	-
9	UM9	-	-	-	-	+	+	+	+	+	-
10	UM10	-	+	-	+	-	+	-	+	+	-

11	UM11	-	-	-	-	-	-	-	+	+	-
12	UM12	+	-	+	-	-	-	-	+	+	-
13	UM13	+	-	-	-	-	-	-	+	-	+
14	UM14	-	+	-	-	-	-	-	+	-	-
15	UM15	-	-	-	-	-	+	+	+	-	-
16	UM16	+	+	-	+	+	+	-	+	-	+
17	UM17	+	-	-	-	-	-	-	+	+	+
18	UM18	-	-	-	-	-	-	-		+	+
19	UM19	-	+	-	-	-	+	-	+	+	+
20	UM20	-	-	-	-	-	-	-	+	+	-
21	UM21	+	-	-	+	-	-	-	+	+	-
22	UM22	-	-	-	-	-	-	-	+	+	-
23	UM23	-	-	-	-	-	+	-	+	-	-
24	UM24	-	+	-	-	+	+	-	+	-	-
25	UM25	-	-	-	-	+	+	-		-	-
26	UM26	+	-	-	-	+	+	-	+	-	+
27	UM27	-	-	-	+	-	+	-	+	+	-
28	UM28	-	-	-	-	-	+	-	+	+	-
29	UM29	-	-	-	-	-	-	-	+	+	-
30	UM30	-	-	-	-	+	-	-	+	+	-
31	UM31	+	-	-	-	-	-	-	+	+	-
32	UM32	-	-	-	-	-	-	-	+	-	+
33	UM33	-	+	-	-	-	+	-	+	-	-
34	UM34	+	-	-	+	-	+	-	+	+	-
35	UM35	-	-	-	-	-	-	-	+	+	-
36	UM36	-	-	-	-	-	-	-	-	+	-
37	UM37	-	-	+	-	-	-	+	-	+	-

38	UM38	+	+	+	+	-	-	-	+	+	+
39	UM39	+	-	-	-	-	-	-	+	+	-
40	UM40	-	-	-	-	-	+	-	+	+	-
41	UM41	-	+	-	-	-	+	-	+	+	-
42	UM42	-	-	-	-	+	+	-	+	-	-
43	UM43	-	-	-	-	-	-	-	+	-	+
44	UM44	+	-	-	-	-	-	-	+	+	+
45	UM45	-	+	-	-	-	-	+	+	+	-
46	UM46	-	-	-	-	-	-	-	+	+	-
47	UM47	-	-	-	-	-	-	-	-	+	-
48	UM48	-	+	-	-	-	-	-	-	+	-
49	UM49	-	-	-	-	+	+	-	+	+	-
50	UM50	+	-	-	-	+	+	-	+	+	-
51	UM51	-	-	-	+	-	+	-	+	+	+
52	UM52	-	+	-	-	-	-	-	+	+	-
53	UM53	-	-	-	-	-	-	-	+	+	-
54	UM54	-	-	-	-	-	-	+	-	-	-
55	UM55	+	-	-	-	-	-	-	+	-	-
56	UM56	+	+	+	-	-	+	-	+	+	+
57	UM57	+	-	-	-	-	+	-	+	+	-
58	UM58	+	+	-	+	+	+	-	+	-	+
59	UM59	-	+	-	+	-	-	-	+	+	-
60	UM60	-	+	-	-	+	-	-	+	+	-
61	UM61	-	-	-	-	+	-	-	-	+	-
62	UM62	+	-	-	-	-	+	-	-	+	-
63	UM63	-	-	-	-	-	-	-	+	+	-
64	UM64	-	-	-	-	-	-	+	+	+	-

65	UM65	+	-	-	-	-	+	-	+	+	+
66	UM66	-	+	-	-	-	+	-	+	+	+
67	UM67	-	-	-	-	-	-	-			+
68	UM68	+	-	-	-	-	-	-	-	-	-
69	UM69	+	-	-	+	-	-	-		+	-
70	UM70	-	-	-	-	-	+	-	+	+	-
71	UM71	-	+	-	-	-	+	-	+	+	-
72	UM72	-	-	-	-	+	+	-	+	+	-
73	UM73	-	-	-	-	-	-	-	+	+	-
74	UM74	+	+	-	-	-	-	+	-	+	-
75	UM75	-	-	-	-	-	-	-	+	+	-
76	UM76	-	+	-	-	-	+	-	+	-	+
77	UM77	-	-	-	+	-	-	-	+	-	-
78	UM78	+	-	-	-	-	-	-	-	-	-
79	UM79	+	-	-	-	-	-	-	-	+	-
80	UM80	-	+	-	-	-	+	-	+	+	-
81	UM81	-	-	-	-	-	+	-	+	+	-
82	UM82	-	-	-	-	-	+	-	+	+	-
83	UM83	+	+	-	+	+	+	-	+	-s	+
84	UM84	+	+	+	-	+	-	-	-	+	-
85	UM85	-	-	-	-	-	-	-	-	+	-
86	UM86	-	-	-	-	-	+	-	+	+	-
87	UM87	-	+	-	-	-	+	-	+	+	-
88	UM88	-	-	-	-	-	-	-	+	+	-
89	UM89	+	-	-	-	-	-	-	+	+	-
90	UM90	-	+	-	-	-	-	-	+	+	-
91	UM91	-	-	-	+	+	+	-	+	-	-

92	UM92	-	-	-	-	+	+	-	+	-	+
93	UM93	+	+	-	-	-	+	-	+	-	+
94	UM94	-	-	-	-	-	+	-	+	-	+
95	UM95	-	+	-	-	-	-	+	-	+	-
96	UM96	-	-	-	-	-	-	-	+	+	-
97	UM97	+	-	-	+	-	+	-	+	+	-
98	UM98	+	+	-	-	-	+	-	+	+	-
99	UM99	+	-	-	-	-	+	-	+	+	-
100	UM100	-	-	-	-	-	+	-	+	+	+
101	UM101	-	+	-	-	+	-	+	+	+	+
102	UM102	-	-	-	-	-	-	-	+	+	+
103	UM103	+	-	-	+	-	-	-	+	+	+
104	UM104	-	+	-	-	-	-	-	+	+	-
105	UM105	-	-	-	-	-	-	-	+	+	-
106	UM106	-	-	-	-	-	+	-	+	+	-
107	UM107	-	-	-	-	-	+	-	+	+	-
108	UM108	+	+	-	-	-	-	-	-	+	-
109	UM109	+	-	-	-	-	-	-	+	+	+
110	UM110	-	-	-	-	+	-	-	+	+	-
111	UM111	-	+	-	+	-	+	-	+	-	-
112	UM112	-	-	-	-	-	+	+	+	-	-
113	UM113	+	-	-	-	-	+	-	+	+	-
114	UM114	-	-	-	-	-	-	-	-	+	+
115	UM115	-	+	-	-	-	-	-	-	+	+
116	UM116	-	-	-	+	-	-	-	-	+	-
117	UM117	+	-	-	+	+	+	-	+	+	-
118	UM118	-	+	-	-	-	-	-	+	+	-

119	UM119	-	-	-	-	-	-	-	+	+	-
120	UM120	-	-	-	-	-	-	-	+	+	-
121	UM121	+	+	-	-	-	+	-	+	+	+
122	UM122	+	+	+	-	-	+	-	+	+	-
123	UM123	-	+	-	-	-	+	+	+	-	-
124	UM124	-	-	-	+	-	+	-	-	-	-
125	UM125	-	-	-	-	-	-	-	-	+	-
126	UM126	-	+	-	-	-	-	-	-	+	-
127	UM127	+	-	-	-	+	+	-	-	+	-
128	UM128	-	-	-	-	+	+	-	+	+	+
129	UM129	-	+	-	-	-	+	-	+	+	+
130	UM130	+	+	-	-	-	+	-	+	+	-
131	UM131	-	-	-	-	-	-	+	+	-	-
132	UM132	-	-	-	+	-	-	-	+	+	-
133	UM133	-	-	-	-	-	-	-	-	+	-
134	UM134	-	+	-	-	+	+	-	+	+	-
135	UM135	+	-	-	-	-	+	-	+	+	-
136	UM136	+	-	-	-	-	+	+	+	+	-
137	UM137	-	-	-	-	+	+	-	+	+	+
138	UM138	-	+	-	-	-	-	-	+	-	-
139	UM139	-	-	-	-	-	-	-	+	+	-
140	UM140	+	+	-	-	-	-	-	+	+	+
141	UM141	-	-	-	+	-	+	+	+	+	-
